# Genetic basis of geographical differentiation and breeding selection for wheat plant architecture traits

**DOI:** 10.1186/s13059-023-02932-x

**Published:** 2023-05-12

**Authors:** Yangyang Liu, Kuocheng Shen, Changbin Yin, Xiaowan Xu, Xuchang Yu, Botao Ye, Zhiwen Sun, Jiayu Dong, Aoyue Bi, Xuebo Zhao, Daxing Xu, Zhonghu He, Xueyong Zhang, Chenyang Hao, Jianhui Wu, Ziying Wang, He Wu, Danni Liu, Lili Zhang, Liping Shen, Yuanfeng Hao, Fei Lu, Zifeng Guo

**Affiliations:** 1 Key Laboratory of Plant Molecular Physiology, Institute of Botany, Chinese Academy of Sciences, Beijing, 100093 China; 2 University of Chinese Academy of Sciences, 100049 Beijing, China; 3 State Key Laboratory of Plant Cell and Chromosome Engineering, Institute of Genetics and Developmental Biology, Innovative Academy of Seed Design, Chinese Academy of Sciences, Beijing, 100010 China; 4 Institute of Crop Sciences, Chinese Academy of Agricultural Sciences (CAAS), Beijing, 100081 China; 5 International Maize and Wheat Improvement Center (CIMMYT) China Office, c/o CAAS, Beijing, 100081, China; 6 State Key Laboratory of Crop Stress Biology for Arid Areas, Northwest A&F University, Yangling, Shaanxi 712100 China; 7 CAS-JIC Centre of Excellence for Plant and Microbial Science (CEPAMS), Institute of Genetics and Developmental Biology, Chinese Academy of Sciences, Beijing, China

**Keywords:** Breeding selection, Geographical differentiation, GWAS, Internode length, Wheat

## Abstract

**Background:**

Plant architecture associated with increased grain yield and adaptation to the local environments is selected during wheat (*Triticum aestivum*) breeding. The internode length of individual stems and tiller length of individual plants are important for the determination of plant architecture. However, few studies have explored the genetic basis of these traits.

**Results:**

Here, we conduct a genome-wide association study (GWAS) to dissect the genetic basis of geographical differentiation of these traits in 306 worldwide wheat accessions including both landraces and traditional varieties. We determine the changes of haplotypes for the associated genomic regions in frequency in 831 wheat accessions that are either introduced from other countries or developed in China from last two decades. We identify 83 loci that are associated with one trait, while the remaining 247 loci are pleiotropic. We also find 163 associated loci are under strong selective sweep. GWAS results demonstrate independent regulation of internode length of individual stems and consistent regulation of tiller length of individual plants. This makes it possible to obtain ideal haplotype combinations of the length of four internodes. We also find that the geographical distribution of the haplotypes explains the observed differences in internode length among the worldwide wheat accessions.

**Conclusion:**

This study provides insights into the genetic basis of plant architecture. It will facilitate gene functional analysis and molecular design of plant architecture for breeding.

**Supplementary Information:**

The online version contains supplementary material available at 10.1186/s13059-023-02932-x.

## Background

Wheat (*Triticum aestivum *L) is a widely cultivated crop on over 200 million hectares with an annual production of approximately 700 million metric tons of grain (http://www.fao.org/faostat/). Wheat contributes to nearly 20% of the total dietary calories and protein consumed worldwide [1]. Raising grain yield remains the main target in wheat breeding. Manipulating plant architecture offers an important approach for the improvement of grain yield in crops. Plant architecture encompasses branching (tillering) pattern, plant height, the shape, size, location of leaves, and reproductive organs. Plant architecture is closely associated with the adaptability of a crop to variable environments, assimilate accumulation, and harvest indexing [2]. According to Donald [3], a crop ideotype is a weak competitor and makes a minimum demand on resources per unit of dry matter produced. An ideal plant architecture is of high efficiency relative to its environmental resources. Ideal plant architecture/node length of wheat is different in variable environments (e.g., drought, lodging). Dwarfing and semi-dwarfing alleles of *Reduced height* (*Rht*) loci substantially reduce plant height and improve assimilate partitioning to spike and high lodging resistance, all further leading to the improvement of grain yields in wheat [4]. In wheat, plant height is determined by internode number, internode length, and spike length. New internodes are produced until the wheat plants reach the floret initiation stage. After this stage, the increase in stem length mainly reflects internode and spike elongation. Thus, internode initiation rate and internode and spike elongation rate during stem elongation are the major determinants for plant height. The different internodes of an individual stem play variable roles in determining grain yield. Combining the desired phenotype of both apical and basal internodes will be beneficial for increasing grain yield in crops.

Crop domestication and breeding induce natural allelic variations, which determine quantitative trait loci (QTLs) associated with agricultural traits. Understanding the genetic basis of phenotypic variation in various germplasm is critical for making accurate selection decisions and for combining desired allelic combinations, which will lead to the improvement of wheat grain yield. Genome-wide association studies (GWAS) offer a powerful approach for dissecting the genetic basis of complex traits and identifying causal polymorphisms [5, 6]. Although a substantial number of wheat GWAS have been reported, these studies are underpowered owing to the relatively small number of single-nucleotide polymorphisms (SNPs) used (< 1million). With the availability of the wheat reference genome and next-generation sequencing technologies for resequencing analyses, comparative genomic sequence analyses enable us to identify more than 100 million SNPs [7–14]. Haplotype analysis of the associated genomic regions may reveal the selection process of the preferred haplotype during wheat breeding. The identification and utilization of superior alleles for the associated traits may greatly facilitate the breeding of new wheat cultivars with high grain yield. The utilization of preferred haplotypes holds great potential for the improvement of wheat grain yield.

To look for the regulators that separately control the length of each internode, and assess the genetic basis of geographical differentiation and breeding selection for wheat plant architecture traits, we conducted GWAS of eight plant architecture traits in 306 worldwide wheat accessions and determined the associated haplotypes and their geographical distribution. We further examined the breeding effects on these haplotypes in 831 wheat accessions that were introduced from other countries or developed in China from 1900 to 2020. Moreover, we explored a number of loci that can separately control the length of internodes within individual stems.

## Results

### Overview of plant architecture traits in this study

**Fig. 1:**
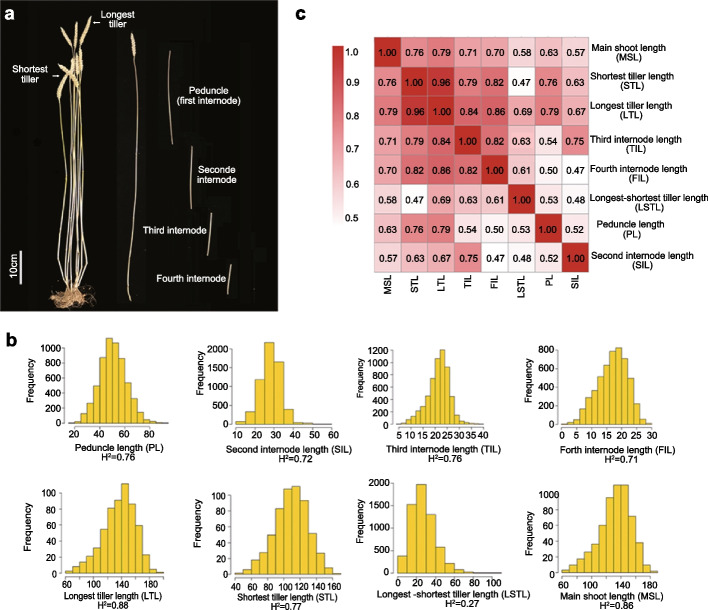
Phenotypic analyses of wheat accessions for eight plant architecture traits. **a** Overview of the eight traits in wheat. The shortest and longest tillers were selected from fertile tillers with spikes. The length of the main shoot as the length of the strongest tiller. The difference between the length of the shortest and longest tillers (longest-shortest tiller) was calculated for each plant. **b** Distribution of phenotypic values and broad sense heritability of the eight plant architecture traits. The broad sense heritability was estimated from the repeatability between raw phenotypes. **c** Pearson’s correlation coefficients were calculated using the phenotypic values for the 306 worldwide wheat accessions

In this study, we analyzed eight plant architecture traits in two environments based on a large-scale phenotypic screen comprised of 306 worldwide wheat accessions, originating from more than 70 countries (Additional file 2: Table S1). The 306 accessions included 179 landraces and 38 traditional cultivars (Additional file 2: Table S1). To investigate the population differentiation of wheat accessions across the globe, we performed population structure analyses. The PCA result revealed that most varieties from Middle East, Europe, and Asia could be distinguished by the first principal component (PC1), with an overall gradient from the Middle East to both Europe and Asia (Additional file 1: Fig. S1a), which is consistent with the history of wheat dispersal [15, 16]. The neighbor-joining tree suggested that the 306 worldwide accessions could classify into some clades, which were associated with their geographical distribution (Additional file 1: Fig. S1b). For instance, Asian accessions and some accessions from Middle East and Africa grouped into the same clade (Additional file 1: Fig. S1b). Most European accessions clustered into one single clade (Additional file 1: Fig. S1b). The eight traits analyzed were the lengths of each of the shortest tiller length (STL), the longest tiller length (LTL), the main shoot length (ML), the peduncle length (first internode below spike) (PL), the second internode length (SIL), the third internode length (TIL), the fourth internode length (FIL) and the difference in the length between the shortest and the longest tiller length (LSTL) (Fig. 1a).

All eight traits displayed extensive variation across the 306 wheat accessions (Fig. 1b). These eight traits exhibited close association and obvious differences between the two environments (Additional file 2: Table S2). We assessed broad sense heritability by calculating repeatability between raw phenotypes (Fig. 1b). Most of the traits demonstrated relatively high heritability (0.71–0.88), while the difference in length between the longest and the shortest tiller exhibited a lower heritability (0.27) (Fig. 1b).

The high-resolution dissection of traits related to plant architecture captured several novel relationships (Fig. 1c). For instance, the lengths of the longest tiller, the shortest tiller, and the main shoot were strongly and positively correlated (*R* ≥ 0.76). This observation suggests the consistency of the longest tiller, the shortest tiller, and the main shoot within individual plants. Peduncle length was strongly and positively associated with the length of the longest tiller (*R* = 0.79) and the shortest tiller (*R* = 0.76), but showed a relatively weak correlation with the length of the other three internodes (*R* = 0.50–0.54). However, the length of the third internode strongly correlated with the length of the fourth internode (*R* = 0.82). The main shoot is the strongest tiller, which is the same to the longest tiller in some cases. This may partially explain the strong correlation (*R* = 0.79) between the length of main shoot and longest tiller.

### Genome-wide association studies reveal shared and independent genetic determinants of plant architecture traits

**Fig. 2:**
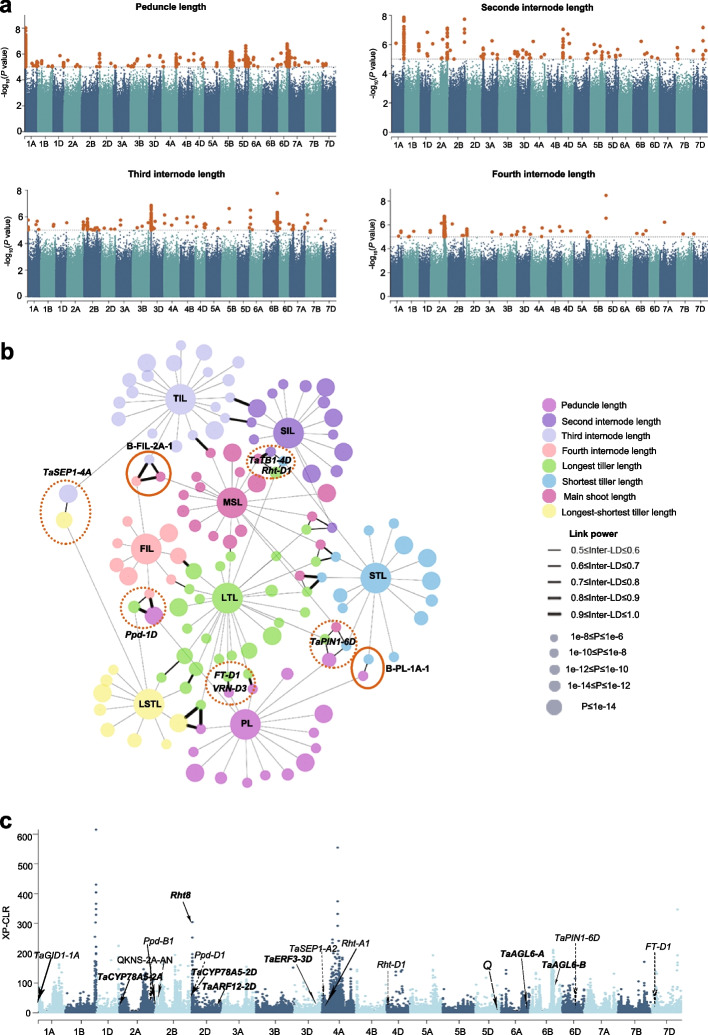
GWAS and network analysis of tiller height across a panel of wheat accessions. **a** Manhattan plots showing the SNP marker-trait associations for the length of the peduncle, the second internode, the third internode, and the fourth internodes. Orange dots indicate SNPs above the significance threshold (− Log_10_[*P-*value] = 5.0). **b** Association networks across different traits in wheat. The nodes represent plant architecture traits and their associated loci. Longest tiller length, LTL; shortest tiller length, STL; length difference between longest and shortest tiller; longest tiller-shortest tiller length LSTL; main shoot length, MSL; peduncle length, PL; second internode length, SIL; third internode length, TIL; fourth internode length, FIL. The eight traits are indicated by different colors. The edges between the loci from different traits are linked by their LD. Only the edges with an average LD ≥ 0.5 are displayed. The orange solid circles indicate the four loci that are specifically associated with the length of the two internodes. The overlapping loci covering *Rht-D1*, *TaPIN1-6D*, *Ppd-D1*, and *TaTB1-4D* are indicated by the orange dashed circles. **c** Distribution of XP-CLR scores (Chinese landraces versus cultivars) for 21 wheat chromosomes. The selected regions and candidate genes detected based on their *p* ratio are shown. The genome-wide threshold was defined by the top 5% of values

In this study, we used genotypic data of wheat accessions from the whole-genome genetic variation map of wheat (VMap [7, 14]). The latest version of VMap (VMap 2.0) [17] consists of 1062 wheat accessions with multiple ploidy levels, from which we selected 306 hexaploid wheat accessions worldwide for this study. The high coverage whole-genome sequencing (~ 10 ×) enabled the identification of 40,710,923 filtered SNPs with minor allele frequency (MAF) > 0.05 across the 306 wheat accessions. Based on these 40,710,923 SNPs, we performed GWAS for the phenotypic values of the eight plant architecture traits in each of the two environments (Y1, 2) and their BLUE values and identified 56,096 (Y1:20,877; Y2:23,315; BLUE:11,904) significant marker–trait associations (− Log_10_ (*P*-value) > 5.0) (Fig. 2a, Additional file 2: Table S3). We used linkage disequilibrium (LD) and connections between markers to delineate about 330 significantly associated loci, when at least five nearby SNPs were above the significance threshold, associated with at least one of the eight plant architecture traits (Additional file 2: Table S4). Of the 330 loci, 83 were associated with a single trait, while the remaining 247 showed pleotropic effects on more than one plant architecture traits (Additional file 2: Table S4). Notably, we observed that some significant loci were specially associated with the length of the four internodes, suggesting the relative independence of the genetic control of internode length in this study.

Pleiotropy and LD play important roles in validating phenotypic correlations [18]. Of the 247 loci with pleotropic effects, 182 were associated with more than four traits, and 64 loci had associations with two or three traits (Fig. 2b, Additional file 2: Table S4). These results suggest that these traits might be genetically co-regulated. We took *TEOSINTE BRANCHED1* (*TB1*) as a control, because recent work reported that *TB1* regulates height and stem internode length in bread wheat [19]. The genomic region (Chr4D: 15,938,883-19,054,796) including *TB1* was associated with the length of second internode, third internode, shortest tiller, longest tiller, and main shoot (Additional file 2: Table S5), suggesting its potential connection with internode length, which is consistent with previous finding [19]. Notably, the major loci for the length of the main shoot (Y1-MSL-1A-1; 2,891 associated SNPs; B-MSL-1A-1:391 associated SNPs), the longest tiller (Y1-LTL-1A-1; 6,079 associated SNPs; B-LTL-1A-1: 27 associated SNPs), the shortest tiller (Y1-STL-1A-1: 1002 associated SNPs; B-STL-1A-1: 5 associated SNPSs), and the peduncle (Y1-PL-1A-1: 5,768 associated SNPs; B-PL-1A-1: 396 associated SNPS; Y2-PL-1A-1: 4 associated SNPs) located in chromosome 1A: 45,791,400-49,172,693 (Additional file 2: Table S4). Most loci for the length of the longest and the shortest tillers overlapped, thus explaining the strong phenotypic correlations between peduncle length and tiller length as well as the consistency of the length among the tillers of individual plants.

To confirm the selected loci in the wheat genome during the gradual improvement of grain yield in China, we selected 59 Chinese wheat accessions (17 cultivars versus 42 landraces) from the 306 wheat accessions for further analysis. These 59 Chinese accessions were selected to display the genomic selection during Chinese wheat breeding process, which will be further used to examine the selection of the associated peaks identified by GWAS. The 59 Chinese accessions are important varieties during Chinese wheat breeding process. We combined the results of whole-genome differentiation of the cross-population composite likelihood ratio (XP-CLR) (Fig. 2c, Additional file 2: Table S6). In total, we determined that 49% of the detected loci (163 of 330) during this study were significantly selected in wheat improvement for higher yield (Fig. 2c, Additional file 2: Table S4). The loci associated with the length of the main shoot, the second internode length, and the longest and shortest tiller in this study (e.g., Y1-MSL-2A-7, Y2-MSL-6B-1, Y2-SIL-2A-1, Y1-SIL-2A-2,, Y2-STL-2A-1, and Y1-LTL-2A-1) as well as known plant height genes (e.g., *Rht-D1,* and *Rht8*) appeared to have experienced a strong selective sweep (Additional file 2: Tables S4 and S6). Moreover, the loci that are specifically associated with the length of the peduncle, and the third and fourth internodes (Y1-PL-1A-1 B-PL-1A-1, chr1A:45791400-49094709; Y1-TIL-3D-1, chr3D:35786271-39382820; Y1-FIL-2A-3, chr2A:613558313-619417320) were among the most selected genomic regions (Fig. 2c, Additional file 2: Table S4). Strong selection for these loci reflected the modifications of plant height traits over the past several decades in wheat breeding. In addition, several candidate genes (e.g., *TaPIN1-6D*, *Ppd-D1*, *SEP1/2-4A,* and *Rht-D1*) associated with spike development, and grain number and size were under strong selection (Additional file 2: Table S6). This result reflected the improvement of adaptation to local environments and grain yield in wheat breeding.

### Genetic basis of geographical differentiation and breeding selection of internode length

The internodes within individual stems play different roles in determining grain yield in crops. To search for loci that can separately regulate the length of the four internodes and determine the genomic basis of geographical differentiation and breeding selection for these traits, we examined the distribution of internode length as a function of the geographical provenance of each accession. We then examined the proportion of each haplotype in all geographical regions based on the four major loci specifically associated with the length of each of the four internodes (Fig. 2a, Additional file 2: Table S1) in the 306 worldwide wheat accessions. In addition, we characterized the extent and direction of the changes of haplotype composition in the 831 wheat accessions (most of the 831 Chinese accessions are modern cultivars) that were introduced from other countries or developed in China since 1900 (Additional file 2: Table S7). The genetic control of agronomical traits might be best understood by comparing a progenitor organism with its derivatives. Genetic crosses between progenitor and derivatives would identify the genetic factors that accounted for their different phenotypes. Thus, we examined the haplotype distribution in the wheat accessions in the two pedigrees between the 1920s and 1970s in China.

#### Peduncle length

**Fig. 3:**
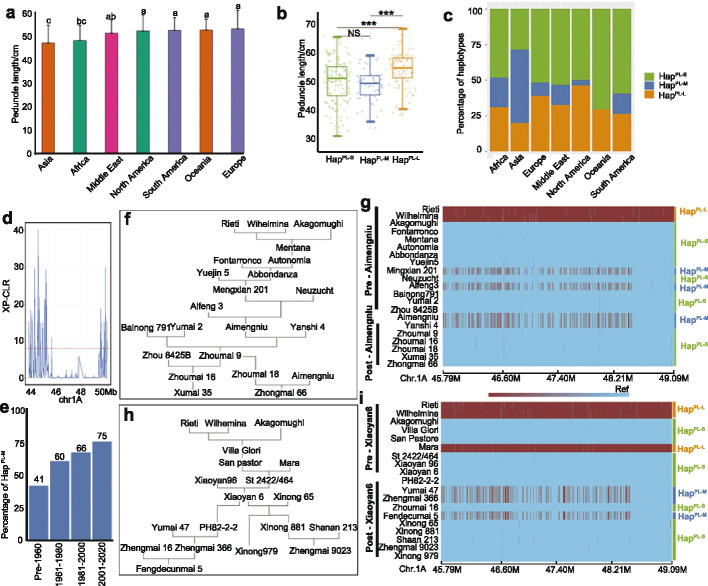
Geographical distribution and breeding selection of the haplotype blocks associated with peduncle length on chromosome 1A. **a** Peduncle length across wheat accessions originating from the seven continents/regions. Data are means ± standard deviation (SD, *n* = 5). Significant differences were determined by ANOVA. Different lowercase letters indicate significant differences (*P* < 0.05). **b** Phenotypic distribution for peduncle length as a function of the three haplotypes on chromosome 1A. Data are means ± SD (*n* = 5). Significant differences were determined by Student’s *t* test (two sided, *P < 0.05, *** *P* < 0.001). **c** The percentages of the three haplotypes for each area. Asia means the region excluding the Middle East. **d** Comparison of XP-CLR score selection signals between Chinese landraces and cultivars for the major locus (Y1-PL-1A-1, chr1A:45,791,400-49,094,709) associated with peduncle length. The red dotted line indicates the threshold (top 5% of scores). **e** Proportion of accessions harboring the haplotype Hap^PL−M^ in four time windows (pre-1960, 1961–1980, 1981–2000, and 2001–2020) in the 831 wheat accessions released between 1900 and 2020 in China. **f**, **h** Pedigree relationships and genomic contribution of founder genotypes to new derived cultivars. In each pedigree, three major global genotypes (Rieti, Wilhelmina, and Akagomughi) contribute to derived cultivars including Aimengniu and Xiaoyan6. The basal rows represent the contribution of Aimengniu and Xiaoyan6 to their derivatives. **g**, **i** Distribution of haplotype blocks for the locus (Y1-PL-1A-1, chr1A:45791400-49094709) in two wheat pedigrees. Each row is a wheat accession and each column is a haplotype. Alleles that are identical to or different from that in the Chinese Spring genome are indicated by blue and red bars, respectively

Peduncle (the first internode below spike) length showed a distinct distribution pattern across the seven continents/regions (Fig. 3a, Additional file 2: Table S8). European accessions had the longest peduncles (52.88 cm), whereas Asian accessions had the shortest peduncles (46.83 cm) (Fig. 3a, Additional file 2: Table S8). The major locus (Y1-PL-1A-1(5768 associated SNPs); B-PL-1A-1(396 associated SNPs); Y2-PL-1A-1 (four associated SNPs) associated with peduncle length mapped to chromosome 1A:45,791,400-49,094,709. We identified three haplotypes of this locus (peduncle length-long, PL-L; peduncle length-medium, PL-M; peduncle length-short, PL-S) in the 306 worldwide wheat accessions: Hap^PL−S^ = 49.27 cm (48.37% of accessions), Hap^PL−M^ = 48.83 cm (20.26% of accessions), Hap^PL−L^ = 53.84 cm (31.37% of accessions) (Fig. 3b, c, Additional file 2: Tables S9 and S10). European accessions exhibited a higher proportion of Hap^PL−L^, whereas Hap^PL−S^ and Hap^PL−M^ was more heavily represented in Asian accessions (Fig. 3b, c, Additional file 2: Table S10). In addition, within each of the three haplotypes, the European accessions always had a longer peduncle than African accessions (Additional file 2: Table S10). These results revealed the genetic basis of the differences in peduncle length between geographical areas.

The genomic region covering Y1-PL-1A-1 was under clear selection between Chinese landraces and cultivars (Fig. 3d). To further assess the selection of Y1-PL-1A-1 in Chinese wheat accessions over the past 120 years, we determined the proportion of each haplotype in 831 wheat accessions that were introduced from other countries or developed in China since 1900 (Fig. 3e, Additional file 2: Table S7). In the 831 wheat accessions, Hap^PL−M^ was the major haplotype (67%, 553 of 831 accessions), whose relative frequency increased from 41% before 1960 to 75% in the time window of 2001–2020. The other two haplotypes, Hap^PL−S^ and Hap^PL−L^, were present in the 200 and 15 Chinese wheat accessions, respectively, indicating that the haplotype Hap^PL−L^ has not been widely used in China. In agreement with this observation, plants including peduncles have become shorter in China over the past 120 years. The dynamics in haplotypes reflected the genomic scale of breeding selection in China over the past 120 years.

To confirm the changes in frequency for the haplotypes at Y1-PL-1A-1, we used two important pedigrees (Aimengniu, Xiaoyan6) in Chinese wheat breeding. We examined the breeding histories of two important cultivars (Aimengniu, Xiaoyan6) that were initially released in the early 1980s. Aimengniu is a high yield variety, which is the founder genotype for more than 20 released Chinese cultivars, many of which are widely planted in China [9]. Xiaoyan6 was recognized through an award in 1985, and more than 40 Chinese cultivars have been derived from this founder genotype [9]. Our collection included a variety of pedigree contributors and subsequently released derived cultivars for both Aimengniu and Xiaoyan6 founder genotypes (Fig. 3f, h). The genetic contribution of founder genotypes to their derived cultivars was reported in our previous work [9]. We examined the distribution of three haplotypes (Hap^PL−S^, Hap^PL−M^, and Hap^PL−L^) in the two pedigrees of two founder genotypes (Aimengniu, Xiaoyan6) (Fig. 3f, h). Both pedigrees were ultimately derived from three major global genotypes (Rieti, Wilhelmina, and Akagomughi) between the 1920s and 1970s [9]. In the first pedigree (Aimengniu), three founder genotypes (Rieti, Wilhelmina, and Akagomughi) contributed to derived cultivars: Mentana, Autonomia, Abbondanza, Mengxian 201, Aimengniu. More recently, Zhoumai 9, Zhoumai 16, Xumai 35, Zhoumai 18, and Zhongmai 66 derived from Aimengniu (Fig. 3f). In addition, some other varieties (e.g., Fontarronco, Yuejin5, Neuzucht, Aifeng5, Bainong791, Yanshi4, Zhou8425B) contributed to derived cultivars in the first pedigree. In the second pedigree (Xiaoyan6), Villa Glori, San Pastore, St 2422/464, and Xiaoyan6 were the derivatives of the same founder genotypes (Rieti, Wilhelmina, and Akagomughi). Next, Xinong 881, Zhengmai 9023, Zhengmai 366, Xinong 979, and Fengdecunmai 5 derived from Xiaoyan6 (Fig. 3h). Moreover, some other varieties (e.g. Mara, Xiaoyan96, Xinong65, Yumai47, Shaan 213, Zhengmai16) contributed to derived cultivars in the second pedigree (Xiaoyan6). All the SNP information of the varieties in the two pedigrees can be obtained from our previous work [9].

The haplotype distribution in the wheat accessions in the pedigrees allowed us to identify the haplotypes that matched the modification of wheat breeding (Fig. 3g, i, Additional file 2: Tables S11 and S12). In the two pedigrees, for the three original founder genotypes, Rieti, Wilhelmina, and Mara harbored Hap^PL−L^, while Akagomughi carried the Hap^PL−S^ (Fig. 3g, i). The remaining derived cultivars had Hap^PL−S^, except Mengxian 201 (first pedigree), Neuzucht (first pedigree), Aimengniu (first pedigree), Yanshi4 (first pedigree), Yumai47 (second pedigree), Zhengmai366 (second pedigree), and Fengdecunmai5 (second pedigree) with Hap^PL−M^ (Fig. 3g, i, Additional file 2: Tables S11 and S12). The different haplotypes of these four derivatives with the three original founder genotypes also indicated the contribution of other founder genotypes. This finding was consistent with reduced plant height in wheat breeding. However, we cannot exclude the possibility that Hap^FIL−S^ was likely introduced from other founders which does not belong to these two pedigrees.

#### Length of the second internode

Oceanian accessions exhibited the longest second internode of all groups (30.11 cm), whereas African accessions had the shortest second internode (26.71 cm) (Additional file 2: Table S8, Additional file 1: Fig. S2a). The major locus (Y1-SIL-1A-2) associated with the length of second internode located to chromosome 1A:555,809,177-557,861,830. We detected two haplotypes at this locus (second internode length-long, SIL-L; second internode length-short, SIL-S) in the 306 worldwide wheat accessions: Hap^SIL−L^ = 28.61 cm (94.44% of accessions), Hap^SIL−S^ = 22.93 cm (4.90% of accessions) (Additional file 2: Tables S9 and S10; Additional file 1: Fig. S2b, c). All Oceanian accessions harbored only Hap^SIL−L^, whereas African accessions presented both Hap^SIL−L^ (89.66%) and Hap^SIL−S^ (10.34%) (Additional file 2: Table S10; Additional file 1: Fig. S2b, c). The distinct distribution of the two haplotypes supported the genomic basis of the phenotypic differences between Oceania and Africa.

The locus Y1-SIL-1A-2 was not a target of selection between Chinese landraces and cultivars (Additional file 2: Table S4; Additional file 1: Fig. S2d). Indeed, we only detected one haplotype (Hap^SIL−L^) across the 831 wheat accessions, making Hap^SIL−S^ absent from Chinese wheat accessions (Additional file 1: Fig. S2e). We examined the haplotype distribution in the two pedigrees, as shown in Fig. 3e, g (Additional file 2: Tables S11 and S12; Additional file 1: Fig. S2f, g). For the three original founder genotypes, Rieti and Wilhelmina carried Hap^SIL−L^, and Akagomughi harbored the Hap^SIL−S^. Importantly, no derivatives in either pedigrees retained Hap^SIL−S^, except Yuejin5 (first pedigree) and Zhou8425B (first pedigree), indicating that Hap^SIL−S^ was selected against these derivatives (Additional file 2: Tables S11 and S12; Additional file 1: Fig. S2f, g), in agreement with its absence in the 831 wheat accessions.

#### Length of the third internode

Middle Eastern accessions had the longest third internode (23.08 cm), whereas Oceanian accessions exhibited the shortest third internode (19.69 cm) (Additional file 2: Table S8; Additional file 1: Fig. S3a). The major locus (Y1-TIL-3D-1) associated with third internode length mapped to chromosome 3D:35,786,271-39,382,820. We identified two haplotypes at this locus (third internode length-long, TIL-L; third internode length-short, TIL-S) in the 306 worldwide wheat accessions: Hap^TIL−S^ = 21.57 cm (94.10% of accessions), Hap^TIL−L^ = 21.87 cm (5.90% of accessions) (Additional file 2: Tables S9 and S10; Additional file 1: Fig. S3b, c). Although we did not observed significant difference of the BLUE values of data in 2021 and 2022, the phenotypic values for these two haplotypes were obviously different for each year (Additional file 2: Table S13). Middle Eastern accessions had the (88.10% of accessions) and Hap^TIL−L^ (11.90% of accessions) Hap^TIL−S^ haplotype, whereas African accessions presented both Hap^TIL−S^ (93.10% of accessions) and Hap^TIL−L^ (6.90% of accessions) (Additional file 2: Table S10, Additional file 1: Fig. S3b, c). The differences of the distribution of the two haplotypes indicated the genomic basis of the phenotypic differences between Africa and the Middle East.

We determined that Y1-TIL-3D-1 was selected between Chinese landraces and cultivars (Additional file 2: Table S4, Additional file 1: Fig. S3d). Indeed, the frequency distribution of Y1-TIL-3D-1 haplotypes changed over time. While Hap^TIL−S^ was the major haplotype in the 831 wheat accessions, its frequency increased from 90% before 1960 phase to 96% after 2000 (Additional file 2: Table S7; Additional file 1: Fig. S3e). In the two pedigrees, all the wheat accessions had Hap^TIL−S^ (Additional file 2: Tables S11 and S12; Additional file 1: Fig. S3f, g).

#### Length of the fourth internode

Middle Eastern accessions had the longest fourth internode (18.23 cm), whereas African accessions had the shortest fourth internode (14.52 cm) (Additional file 2: Table S8; Additional file 1: Fig. S4a). The major locus (Y1-FIL-2A-3; B-FIL-2A-1) associated with fourth internode length located to chromosome 2A:613,558,313–619,417,320. We detected two haplotypes at this locus (fourth internode length-long, FIL-L; fourth internode length-short, FIL-S) in the 306 worldwide wheat accessions: Hap^FIL−L^ = 17.54 cm (90.82%), Hap^FIL−S^ = 11.69 cm (9.18%) (Additional file 2: Tables S9 and S10; Additional file 1: Fig. S4b, c). All Middle Eastern accessions harbored Hap^TIL−S^, whereas African accessions included both Hap^FIL−S^ (6.90%) and Hap^FIL−L^ (93.10%) (Additional file 2: Table S10; Additional file 1: Fig. S4b, c), the distribution differences of the two haplotypes indicated the genomic basis of the phenotypic differences between Africa and the Middle East.

Y1-FIL-2A-3 and B-FIL-2A-1 were clearly selected between Chinese landraces and cultivars (Additional file 2: Table S4; Additional file 1: Fig. S4d). The two haplotypes at Y1-FIL-2A-3 displayed changes in their frequencies across breeding periods. In the 831 wheat accessions, Hap^FIL−L^ was the major haplotype in 72% of accessions released prior to 1960, this percentage decreased from 72% before 1960 to 37% after 2000 (Additional file 2: Table S7; Additional file 1: Fig. S4e). In the two pedigrees, all the three founder genotypes (Rieti, Wilhelmina, and Akagomughi) harbored Hap^FIL−L^. In the first pedigree (Aimengniu), most derived cultivars carried Hap^FIL−S^, four derived cultivars (i.e. Neuzucht, Yumai2, Zhou8425B, Yanshi4) had Hap^FIL−L^ (Additional file 2: Table S11; Additional file 1: Fig. S4f). In the second pedigree (Xiaoyan6), Xiaoyan6, PH62-2–2, Zhoumai16, Xinong65, Shaan 213, Zhoumai9023, and Xinong979 had Hap^FIL−S^ (Additional file 2: Table S12; Additional file 1: Fig. S4g). These results suggest that Hap^FIL−S^ was introduced from other founder genotypes, rather than the three founder genotypes (Rieti, Wilhelmina, and Akagomughi).

**Fig. 4:**
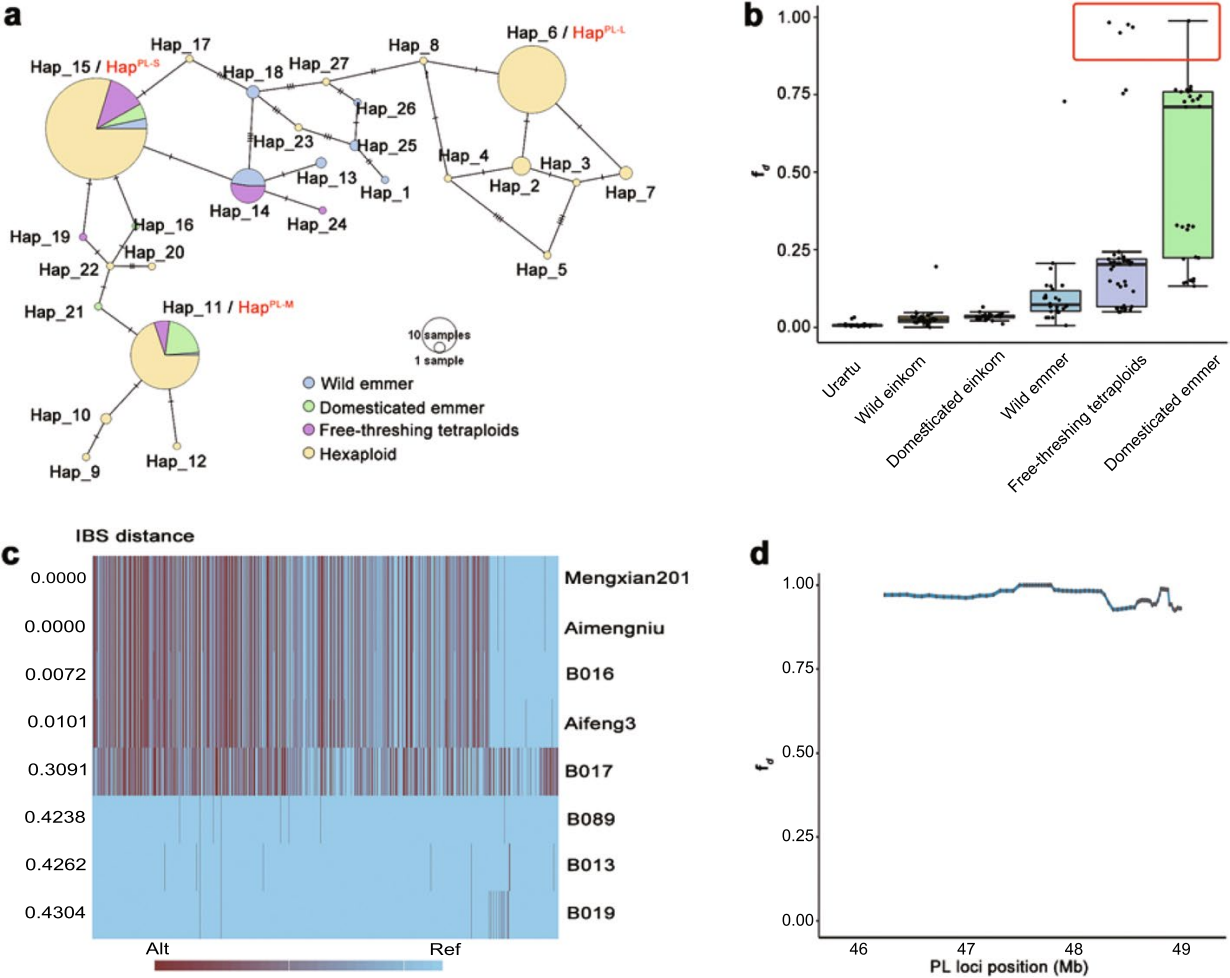
The evolutionary relationship of the haplotypes for Peduncle length (PL) locus (chromosome 1A:45,791,400-49,094,709). **a** Haplotype networks for PL locus. The haplotype networks were developed based on the SNPs in high linkage disequilibrium in a haplotype using the 306 hexaploid wheat accessions, and 126 additional accessions (30 Aegilops tauschii, 96 tetraploid wheat varieties). **b** Introgression from donor populations across PL locus (1A:45,791,400-49,094,709). Mean of *f*_*d*_ was calculated on PL locus using Indian Dwarf Wheat as P1, Mengxian201 as P2, Rye as outgroup in four-taxon topology ((P1, P2), P3, O). **c** Haplotype blocks on PL locus with candidate introgression donors of Mengxian201. **d** The window based *f*_*d*_ value on PL locus when B016 is used as P3, the window is set with 100 SNPs, with stepsize of 5 SNPs calculated using the python script available at https://github.com/simonhmartin/genomics_general

To evaluate the evolutionary relationship of the haplotypes for loci of the length of the four internodes, in addition to the 306 hexaploid wheat accessions, we used 126 additional accessions including 30 Aegilops tauschii, 96 tetraploid wheat varieties, which have been sequenced previously [7]. We extracted SNPs that are in high linkage disequilibrium in a haplotype and used popArt [20] to visualize the network (Fig. 4a; Additional file 2: Table S15; Additional file 1: Fig. S5).

The haplotypes of loci for peduncle length (PL) locus (1A:45,791,400-49,094,709), second internode length (SIL, 1A:555,809,177-557,861,830), and fourth internode length (FIL, 2A:613,558,313-619,417,320) appeared to derive from wild emmer (Additional file 1: Fig. S5). In addition, the introgression of these haplotypes from domesticated emmer and free-threshing tetraploids may partly contribute to their appearance in hexaploid wheat (Additional file 1: Fig. S5). This evolution process of these haplotypes for these two loci is consistent with the evolutionary history of hexaploid wheat (Additional file 1: Fig. S5). The haplotypes of locus for third internode length (TIL, 3D:35,786,271-39,382,820) may be infiltrated from Anathera, Meyeri, and Strangulata (Additional file 1: Fig. S5c). Consistently, our previous work indicated that Strangulata is the donor of D genome [7].

The haplotype of PL locus (1A:45,791,400-49,094,709) in Mengxian201 was unclear in the first pedigree (Fig. 3g). We therefore examined the potential genetic donor of haplotypes for Mengxian201. The potential donors are four free-threshing tetraploids and one domesticated emmer indicated by *f*_*d*_ > 0.9 (Fig. 4b). To further seek the donor of Mengxian201, we analyzed the haplotype blocks on this locus with its possible donors, one free-threshing tetraploid B016 turns out to be the most possible donor of Mengxian201, because it has the most similar haplotype blocks with Mengxian201 among all five candidates and *f*_*d*_ values are nearly equal to 1 across the locus (Fig. 4c, d). Furthermore, the minimum IBS distance also support this hypothesis, the value of IBS distance between Mengxian201 and B016 is even smaller than that with the hexaploid landrace Aifeng3 (Fig. 4c). Our work indicated that free-threshing tetraploids B016 is the most possible donor of Mengxian201; however, it might not be the direct donor, since we cannot exclude the other possibilities during wheat breeding, such as natural outcrossing, heterozygous parent.

We observed obvious differences of the phenotypic values between the two environments for the length of four internodes (Additional file 2: Table S2). Notably, the genotypic values for the length of the first and second internodes were higher in 2021 than those in 2022. However, the genotypic values for the length of the third and fourth internodes were higher in 2022 than those in 2021. The Shukla model [21] was used to evaluate the interaction between genotypes and environments (Additional file 2: Table S14). Obvious interactions between haplotypes and environments for four haplotypes (Hap^PL−S^, Hap^SIL−L^, Hap^SIL−S^, Hap^TIL−L^) were observed in this study (Additional file 2: Table S14). In addition, the results suggested the relative stability of other haplotypes of the length of four internodes (Additional file 2: Table S14).

### Genomic basis of geographical differentiation of tiller length

We identified a locus on chromosome 1A between 45,791,400 and 49,172,693 bp that was associated with the length of the longest tiller, the shortest tiller, and the main shoot. This locus mapped to the same position as the major locus of the first internode length mentioned above (Additional file 2: Table S4). We observed the highest values for the longest tiller, shortest tiller, and the main shoot in European wheat accessions and the shortest values in African wheat accessions, which was the same trend as that seen for the length of the first internode (Additional file 2: Table S8). Similarly, the three haplotypes associated with the locus for first internode length displayed identical effects on the length of the longest tiller, shortest tiller, and the main shoot (Additional file 2: Table S10), suggesting that this observed haplotype distribution might explain the geographical distribution of these phenotypes.

### Geographical differentiation and breeding selection of haplotype combinations for the length of the internodes

**Fig. 5:**
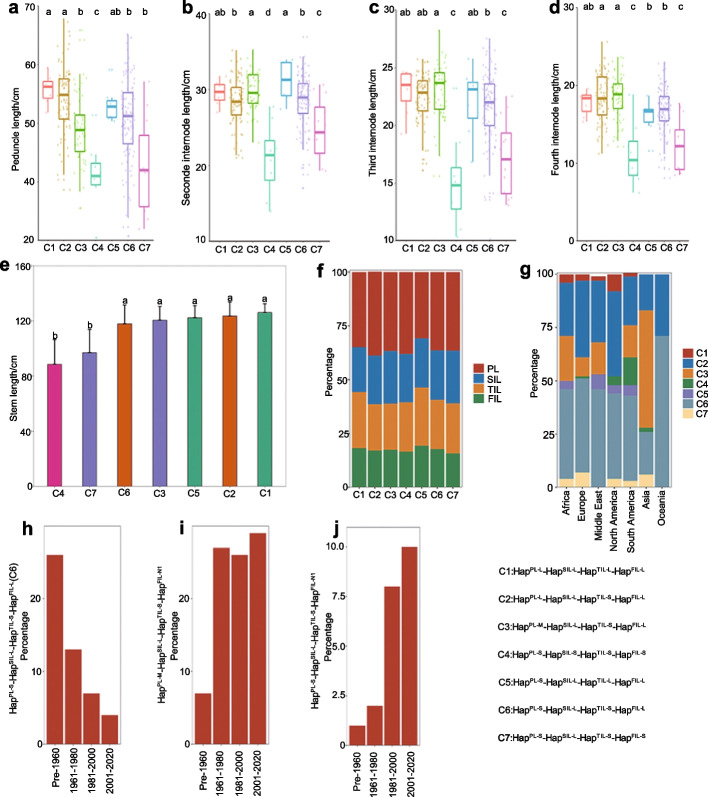
The geographical distribution, breeding selection, and effects of haplotype combinations on associated traits. **a**–**e** The differences of the length of the peduncle (**a**), the second internode (**b**), the third internode (**c**), the fourth internode (**d**), and the stem (**e**) among the seven haplotype combinations. Data are shown as means ± SD (*n* = 5). Significant differences were determined by ANOVA. Different lowercase letters indicate significant differences (*P* < 0.05). **f** The percentages of the length of the peduncle (PL), the second internode (SIL), the third internode (TIL), and the fourth internode (FIL) relative to stem length. **g** Geographical distribution of the seven haplotype combinations for each area. **h**–**j** Frequency of three haplotype combinations among 831 Chinese wheat accessions released from 1900 to 2020. Hap^FIL−N1^ was a new haplotype in 831 Chinese wheat accessions, which was not observed in 306 worldwide wheat accessions

Wheat breeding has exploited variable haplotypes associated with agricultural traits. Understanding the genetic basis of this phenotypic variation in various germplasms is critical for making accurate selection decisions and for combining desired haplotype combinations to improve wheat grain yield. We detected seven major haplotype combinations of the four major loci for the length of the four internodes. These seven combinations accounted for 94.12% (288 accessions) of all the haplotypes in the 306 worldwide wheat accessions (Additional file 2: Table S16). We determined the effects of the seven combinations on the associated traits (Fig. 5a–d, Additional file 2: Table S17). The four internodes were shorter in haplotype combination 4 (C4, Hap^PL−S^-Hap^SIL−S^- Hap^TIL−S^-Hap^FIL−S^) and C7 (Hap^PL−S^-Hap^SIL−L^-Hap^TIL−S^-Hap^FIL−S^) than the other five haplotype combinations (C1, C2, C3, C5, C6), although these five combinations harbored one or two haplotypes that negatively regulate the length of internodes (Fig. 5a–d, Additional file 2: Table S17). This indicated that the haplotypes of negative effects on the length of internodes are synergistic in these seven combinations. Consistently, C4 and C7 exhibited shorter stems compared to the other five combinations (Fig. 5e, Additional file 2: Table S17). As expected, the peduncle length had a higher proportion in the stem length relative to the length of second, third, and fourth internodes (Fig. 5f, Additional file 2: Table S17). More accessions harbored C2 and C6 than other combinations in most areas (Fig. 5g, Additional file 2: Table S17). However, C3 was more widely distributed in Asia (except the Middle East) relative to other areas (Fig. 5g).

We further explored the history of haplotype combinations of the four major loci for the length of the four internodes in 831 Chinese wheat accessions released between 1900 and 2020 (Fig. 5h–j, Additional file 2: Table S18). In the 831 Chinese wheat accessions, we identified 32 haplotype combinations, but did not detect C1 and C4 from the 306 worldwide wheat accessions. Of the 32 combinations, three combinations had been obviously selected in Chinese wheat breeding. C6 of the 306 worldwide wheat accessions was also observed in 831 Chinese wheat accessions. C6 decreased in frequency from 26.05% of all accessions before 1960 to 13.33% (1961–1980), 7.22% (1981–2000), and 3.98% (2001–2020) (Fig. 5h, Additional file 2: Table S18). The haplotype combination (Hap^PL−M^-Hap^SIL−L^-Hap^TIL−S^-Hap^FIL−N1^) increased obviously in frequency from pre-1961 to 1961–1980 among Chinese wheat accessions, suggesting that this combination was selected during this time window (Fig. 5i, Additional file 2: Table S18). Similarly, another haplotype combination (Hap^PL−S^-Hap^SIL−L^-Hap^TIL−S^-Hap^FIL−N1^) in frequency increased from 0.84% of all accessions before 1960 to 2.22% (1961–1980), 7.73% (1981–2000), and 10.30% (2001–2020) (Fig. 5j, Additional file 2: Table S18).

## Discussion

Plant breeders have paid special attention to plant architecture for decades because of its significance for improving varieties. Plant architecture plays a decisive role in grain yield potential. Plants with reduced height benefit from improved lodging resistance and assimilates partitioning to the developing spike, facilitating improved floret fertility and grain numbers per spike [22].

Some QTLs and genes associated with internode length have been identified in different species, which provided genetic resources for the manipulation of plant height through the length of different internodes [23–28]. To the best of our knowledge, in wheat, some genetic work related to peduncle have been reported [29, 30]. However, few genetic studies dissected the length of internodes or the length of the shortest and longest tillers within individual plants. The only publication reported the QTLs of the internode length using two biparental populations without the physical positions in wheat genome, since the information of reference genome was not available at that time [31]. Some identified loci in this study were overlapped with reported QTLs or genes in previous work. Nevertheless, a big proportion of the identified loci, especially these loci associated with four internodes, are novel relative to previous studies.

In this study, we determined the phenotypic variation for eight plant architecture traits, including the length of the four internodes and the shortest and the longest tiller, based on 306 worldwide wheat accessions originating from the seven continents/regions. We used whole-genome sequencing data for these 306 wheat accessions, with an average depth of 10 × coverage and identified about 40 million high-confidence and high-quality SNPs. GWAS results identified QTLs that are independent of the known *Rht* genes, therefore providing new resources for the genetic control of plant height and internode length.

Few studies have explored the genetic basis of the geographical distribution and breeding selection of the internode length. In this study, the worldwide distribution of the haplotypes underlying each of the identified QTLs offers a glimpse into the genomic basis behind the phenotypic differences for the eight traits measured here as a function of geographical origin. In addition, we examined the selection of all haplotypes during wheat over the past 120 years in China. These haplotypes have been clearly selected (for or against) in Chinese breeding, indicating that the observed alterations of haplotypes underscore the genomic basis of modification for plant architecture traits in the past 120 years in China.

**Fig. 6:**
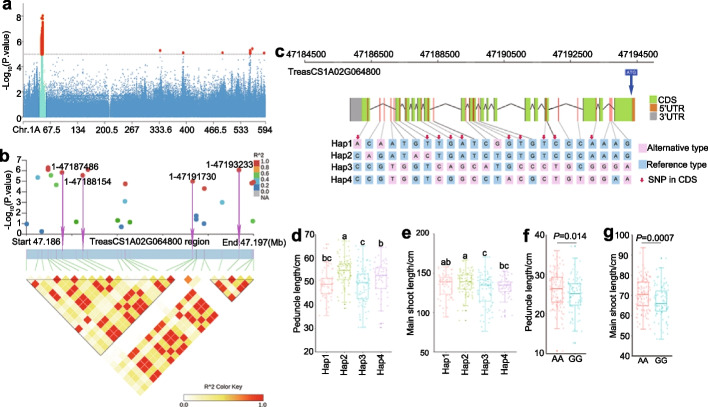
Contributions of *TraesCS1A02G064800* alleles to the length of the peduncle and the main shoot in RILs. **a** The associated signals on chromosome 1A. Red dots indicate the SNPs of the marker-trait associations above the significance threshold (− Log_10_[*P*-value] = 5.0). **b** Pairwise LD analysis. Local Manhattan plot (top) and LD heat map (bottom) surrounding the peak on chromosome 1A. Red color highlights the strong LD with the significant variants. Arrows indicate the four SNPs that were significantly (− Log_10_[*P*-value] > 5.0) associated with the traits and located in the exonic region of this gene *TraesCS1A02G064800*. **c** Gene structure and the haplotypes of *TraesCS1A02G064800*. Green rectangles and black lines indicate exons and introns, respectively. The gene is located on the reverse strand. The haplotypes were determined based on 306 worldwide wheat accessions. The SNPs of the haplotypes were indicated in **c**. **d**, **e** The differences of the length of the peduncle (**d**) and the main shoot (**e**) and among the four haplotypes (*n* = 59 accessions (Hap1), 93 accessions (Hap2), 88 accessions (Hap3), 53 accessions (Hap4) of *TraesCS1A02G064800*. Data are means ± SD (*n* = 5). Significant differences were determined by ANOVA. Different lowercase letters indicate significant differences (*P* < 0.05). **f**, **g** Contrast between original Zhongmai175 alleles (AA, long plants, RIL^long^) and Yanzhan4110 alleles (GG, short plants, RIL^short^) with respect to the length of the peduncle (**f**) and the main shoot (**g**). Data are shown as mean ± SD (*n* = 5). Significant differences were determined by Student’s *t* test (two sided)

The peduncle, the first internode below the spike, plays variable roles in the determination of crop grain yield. The peduncle vascular system is critical for the transport of photosynthetic products, nutrients, and water from the roots and leaves to the filling grain [32, 33]. We identified one major locus related to the length of peduncle and main shoot. Within this major locus, four SNPs were significantly (− Log_10_[*P*-value] > 5.0) associated with the traits and located in the gene *TraesCS1A02G064800*. Therefore, we identified *TraesCS1A02G064800* as a candidate gene that controlled peduncle length. The orthologue of *TraesCS1A02G064800* in Arabidopsis is *Trehalose-6-P synthase 1*. We identified four major haplotypes at *TraesCS1A02G064800* in the 306 worldwide wheat accessions (Fig. 6c, Additional file 2: Table S19). These four haplotypes comprised 59, 93, 88, and 53 accessions, respectively (Additional file 2: Table S19). We determined the effects of the four haplotypes on the associated traits (Fig. 6d, e, Additional file 2: Table S16). There were significant differences in the length of the peduncle (main shoot) and the main shoot between these four haplotypes (Fig. 6d, e, Additional file 2: Table S16). Therefore, we identified *TraesCS1A02G064800* as a candidate gene that controlled the length of peduncle and main shoot.

To compare the contributions of *TraesCS1A02G064800* alleles to the length of peduncle and the main shoot and evaluate its potential value in wheat breeding, we assessed the phenotypic differences of these two traits between the alleles in a genetic population. Sequencing the coding sequence (CDS) of *TraesCS1A02G064800* resulted in the identification of a nonsynonymous A/G SNP (4,245 bp into the gene, 1,046 bp in the coding sequence) between the cultivars Zhongmai175 (AA, average main shoot length of 71.50 cm, average peduncle length of 43.00 cm) and Yanzhan4110 (GG, average main shoot length of 58.50 cm, average peduncle length of 30.50 cm). The nonsynonymous SNP caused a change from aspartate (Zhongmai175) to glutamate (Yanzhan4110). Asian accessions exhibited a higher proportion of the allele with longer main shoot and peduncle (AA, 72.06%), whereas the allele with shorter main shoot and peduncle was more heavily represented in Oceania (GG, 70.59%) (Additional file 2: Table S20). These two alleles were present almost equally in the remaining five areas (Additional file 2: Table S20).

We examined recombinant inbred lines (RILs) generated from the parents Zhongmai175 (AA, RILs^long^) and Yanzhan4110 (GG, RILs^short^). On average, the RILs^long^ (average plant height 70.46 cm, average peduncle length 26.22 cm) had taller plants than the RILs^short^ (average plant height 66.79 cm, average peduncle length 24.84 cm) (Fig. 5g, Additional file 2: Table S21). These results suggested the potential of *TraesCS1A02G064800* alleles in the modification of plant height by regulating peduncle length (Fig. 6f, g).

Lodging is a serious concern that leads to lower grain yield in most cereal crops. The length of basal internodes is closely connected to lodging resistance. *Shortened Basal Internodes* (*SBI*) encodes a gibberellin 2-oxidase and specifically controls the elongation of basal internodes in rice [34]. Similarly, in tomato (*Solanum lycopersicum*), a gene controlling internode elongation was mapped to *GA2oxidase 7*, a class III GA 2-oxidase in the gibberellin biosynthetic pathway [35]. We identified loci associated with the length of basal internodes (the third and fourth internodes) that specifically control the length of basal internodes and not that of other internodes or shoot length. These loci offer the new resources for the improvement of lodging resistance in wheat. The haplotype distribution across different areas worldwide and in various time windows in the past 120 years is consistent with the phenotypic differences, which provides the genomic basis of geographical differentiation and breeding selection for wheat plant architecture traits. Independent control of internode length of individual stems enabled us to get desired haplotype combinations of the length of four internodes with different functions.

The spike number per unit area is one of the key factors that determine grain yield in wheat [36, 37]. The difference in the length between the shortest and the longest tiller reveals the space that can accommodate spikes [38–40]. In this study, we identified 30 loci related to this difference, of which 15 loci (Additional file 2: Table S4) only connected to this trait, suggesting the potential for these loci to manipulate the number of spikes.

## Conclusions

In summary, our work explored the genomic basis of geographical differentiation and breeding selection for wheat plant architecture traits. We revealed the genetic association networks across different traits as well as the loci specifically controlling individual traits. This information will be helpful for the improvement of cultivars during wheat breeding.

## Methods

### Planting and phenotyping

Seeds for 306 worldwide wheat accessions were sown with two replicates to ensure data repeatability in Zhaoxian, Hebei province, China (37° 27′ N, 113° 30′ E, altitude 78 m) in 2020–2021 and 2021–2022. For each replicate, six rows were planted per accession, with each row (1.5 m in length) including 15 plants, with 10 cm between rows. For the sowing, we used a self-made hole punch to ensure the consistency of plant spacing and sowing depth. The punch is welded on a 1-m-long steel plate, on which steel columns with equal spacing are evenly distributed. These steel columns are thick at the top and thin at the bottom and have equal length, which allowed us to ensure that the hole depth is consistent.

The phenotypic data of the eight traits exhibited differences and similarity between the two locations, and closely correlated with each other (Additional file 2: Table S11). All field management tasks (e.g., irrigation, weed management, and fertilization) were performed according to the normal standards. Plants were irrigated when required. Seven traits were measured at physiological maturity, and the resulting values were used to calculate one trait (the difference between the length of the shortest and longest tiller). Five plants were randomly selected as five replicates for each accession to determine the eight traits at each location. The eight traits include the length of the first (peduncle), second, third, and fourth internode from top; the length of the shortest main shoot and the longest main shoot; and the difference between the length of the shortest and the longest tiller. The main shoot was selected as the strongest tiller for each plant. The longest and shortest tillers were determined by the length of fertile tillers with spikes for individual plants. The first, second, third, and fourth internodes were measured along the main shoot from the top of plants.

### Statistical analysis of plant architecture traits

To obtain best linear unbiased predictor (BLUE) values, the values of eight plant architecture traits in all environments were calculated using the *lem4* package in R [41]. The formula was as follows:

$$Y=\mathrm\mu+\mathrm{Line}+\mathrm{Env}+(\mathrm{Line}\times\mathrm{Env})+(\mathrm{Env}\times\mathrm{Rep})+\mathrm{error}$$

where *μ* is the mean, Line is the genotype effect, Env is the environment, Line × Env is the genotype and environment interaction, Env × Rep is the environment and replication interaction, and error is the error of the environment and the replication.

### Genotype calling and SNP identification

SNP discovery and genotyping were performed through a well-established pipeline used for the construction of version 2.0 of the whole-genome genetic variation map of wheat (VMap 2.0) [17]. A total of 306 hexaploid wheat accessions were selected from VMap 2.0 for this study. A total of 40,710,923 segregating SNPs (minimum allele frequency [MAF] > 0.05; missing rate < 20%; missing genotype rate < 10%) were used for GWAS. For the 831 Chinese accessions, we genotyped all the accessions with wheat 660 K SNP array [42]. Genotype imputation is a process of estimating missing genotypes from the reference panel and is commonly performed in GWAS to increase the number of useful markers [43–45]. The SNPs for 831 wheat accessions were obtained by genotype imputation based on the SNPs of 306 worldwide wheat accessions. Genotype imputation was conducted based on the source data according to previous work [43–45]. Out of 831 wheat accessions, 86 were previously resequenced with an average read depth of 17.9 × for each accession [9]. The genome sequence of these 86 wheat accessions was used to check and correct the genotype imputations for the 831 wheat accessions. The source data for the SNPs of the 86 wheat accessions were downloaded from the wheat Union database [46].

### Genome wide association study (GWAS)

The GWAS was performed for eight plant architecture traits on 306 worldwide wheat accessions using 40,710,923 SNPs (MAF > 0.05; missing rate < 20%; missing genotype rate < 10%). The algorithm efficient mixed model association expedited (EMMAX) can efficiently correct for a wide range of population structures, which would otherwise lead to spurious genotype–phenotype associations in a GWAS [47]. Therefore, the GWAS was conducted using GEMMA (version 0.98.4) by fitting the mixed linear model (MLM) association expedited (EMMAX) algorithm, including kinship as a correlation matrix.

The top three principal components (PCs) from principal component analysis (PCA) were used to build the matrix for population structure correction using Plink [48] with the parameters in the program set to “–pca 10”. The matrix of simple matching coefficients was used to build the kinship (*K*) matrix. Genetic relationship between accessions was modeled as a random effect using the *K* matrix. We used significant *P*-value thresholds (*P* < 10^–5^) to control genome-wide type I errors according to previous study that included identical SNP number in wheat [9].

### Linkage disequilibrium analysis

To determine the genomic regions of interest, the SNPs associated with all traits above a significance threshold of −Log_10_(*P*-value) = 5 were combined and the duplicates removed with vcftools. LD between SNPs was calculated by PLINK [48], with the parameters in the program set to “ --allow-no-sex --maf 0.05 --geno 0.2 --r2 --ld-window 50000 --ld-window-r2 0.” The results were used to combine SNPs to define intervals based on the LD between markers, with markers with* r*^*2*^ > 0.1 being included in the same interval. If the distance between the peak SNPs of two adjacent loci was less than 5 Mb, these two loci were merged. The number of significant SNPs contained within each genomic region was counted. If the SNP number in the corresponding genomic region was more than 5, the region was defined as a QTL. The software *LDBlockShow* was used to conduct the pairwise LD analysis of the associated genomic region for *TraesCS1A02G064800* [49].

### Construction of association networks

The analysis of association network was conducted using the software *Cytoscape* [50] (Version: 3.2.1). The network displayed the connections between the traits and their corresponding loci as well as the links between loci (average *r*^*2*^ ≥ 0.5). The effective scores for each locus are represented by *P-*values of the most significant SNPs associated with the corresponding traits in the GWAS. The link between pairs of loci was represented as their average LD. Here, the LD was calculated according to previous work [6] as follows:

$$\mathrm{LD}=1/2\ast(\mathrm{LD}\,(\mathrm{locus}1,\,\mathrm{locus}2)/\mathrm{PmaxLD}(\mathrm{locus}1)+\mathrm{LD}(\mathrm{locusn}1,\,\mathrm{locus}2)/\mathrm{PmaxLD}(\mathrm{locus}2))$$

with LD (locus1, locus2) being the average pairwise LD value (*r*^2^) between all SNPs of locus1 and all SNPs of locus2; PmaxLD(locus1)/PmaxLD(locus2) is the largest possible LD value within the locus1/locus2 locus, obtained by calculating the average *r*^2^ of each SNP against all SNPs from the locus1/locus2 locus. The maximum average LD value represents this locus’s PmaxLD. Pairwise *r*^2^ values were calculated between all significant SNPs using PLINK [48].

### SNP-based haplotype construction for loci

The SNP-based haplotype construction for each locus was evaluated using the *LDheatmap* and *Pheatmap* software package in R. The *Pheatmap* package defines haplotype blocks and provides the number of haplotypes and their physical length (bp) for each block, as well as the number of tagged SNPs. If the SNP was the same as in the reference type (Chinese Spring), it was given a value of 0; if the SNP was different from that of Chinese Spring, it was given a value of 1. Heterozygous and missing SNP were given a value of 0.5. Full cluster analysis was performed on all accessions using Euclidean distance using *Pheatmap* (version1.0.12) in R. The *LDheatmap* package in R [51] was used to conduct LD analysis for each locus in this study.

### Identification of putative selective sweeps

The XP-CLR test [52] was used to detect selective sweeps to identify potential selective signals between Chinese cultivars (17 accessions) and landraces (42 accessions, reference population) in 306 word wheat accessions. The XP-CLR score between two wheat populations was calculated using the parameters “--ld 0.95 --maxsnps 1000 --size 50000 --step 20000.” To detect which gene was under selection, the selection sweeps were ranked based on decreasing XP-CLR scores, and the top 5% regions were chosen as selective sweeps.

### The interaction between genotypes and environments, and the stability of haplotypes

The Shukla model [21] was used to evaluate the interaction between genotypes and environments. The interaction evaluated using the formula as follows:

$${\mathrm{ge}}_{\mathrm{ij}}={\mathrm y}_{\mathrm{ij}}-{\mathrm g}_{\mathrm i}-{\mathrm e}_{\mathrm j}-\mathrm\mu$$

where *ge*_*ij*_ is the interaction between genotypes and environemts, *y*_*ij*_ is the phenotypic values of genotype *i* in environment *j*, *g*_*i*_ is the effects of genotype *i*, *e*_*j*_ is the effects of environment *j*, and *μ* is the mean of the corresponding traits for all the genotypes in all the environments.

### The *f*_d_ statistic analysis

The *f*_*d*_ statistic can estimate the proportion of introgression in a given window [53]. We estimated the *f*_*d*_ values across the genome using the python code available at https://github.com/simonhmartin/genomics_general. The sliding window was set with a window size of 100 SNPs and a step size of 5 SNPs. We converted the *f*_*d*_ statistic value to 0 for windows of *D* < 0 because the negative *f*_*d*_ statistic value is meaningless. We estimated the *f*_*d*_ statistic value using Indian dwarf wheat as P1, Mengxian201 as P2, rye as outgroup in four-taxon topology ((P1, P2), P3, O), the P3 are diploid and tetraploid relatives of bread wheat, including 28 urartu, 31 wild einkorn, 31 domesticated einkorn, 26 wild emmer, 29 domesticated emmer, 41 free-threshing tetraploids.

## Supplementary Information

**Additional file 1: Figure S1.** Phylogenetic relationships and population structures. **Figure S2.** Geographical distribution and breeding selection of the haplotype blocks associated with the length of the second internode on chromosome 1A. **Figure S3.** Geographical distribution and breeding selection of the haplotype blocks associated with the length of the third internode on chromosome 3D. **Figure S4.** Geographical distribution and breeding selection of the haplotype blocks associated with the length of the fourth internode on chromosome 2A. **Figure S5.** The evolutionary relationship of the haplotypes for the loci for the length of second internode, third internode, four internode.

**Additional file 2: Table S1.** Summary of the 306 worldwide wheat accessions. **Table S2.** Comparison of the eight plant architecture traits between two environments. **Table S3.** Detailed information of all significantly associated SNPs for the investigated traits> 5). **Table S4.** The 330 identified loci associated with investigated traits in this study. **Table S5.** Overlap between the known genes/QTLs and 330 identified loci in this study. **Table S6.** XP-CLR scores for the known genes. **Table S7.** The haplotypes of four major loci for the length of the four internodes in 831 Chinese wheat accessions. **Table S8.** The phenotypic data for the varieties from the seven continents/regions. **Table S9.** The phenotypic data of the haplotypes of the four major loci for the length of the four internodes. **Table S10.** The distribution of haplotypes of the four internodes in 306 worldwide accessions. **Table S11.** The genotypic data of all the varieties in the first pedigree. **Table S12.** The genotypic data of all the varieties in the second pedigree. **Table S13.** The interaction between environments and haplotypes for the length of the four internodes. **Table S14.** The interaction between environments and haplotypes for the length of the four internodes. **Table S15.** The 432 wheat varieties used for the analysis of the evolutionary relationship of the haplotypes. **Table S16.** The haplotype combinations for the length of the four internodes in 306 worldwide wheat accessions. **Table S17.** The haplotype combinations for the length of the four internodes in 306 worldwide wheat accessions. **Table S18.** The distribution of haplotype combinations for the length of the four internodes in 831 Chinese wheat accessions. **Table S19.** The phenotypic data of the four major haplotypes of TraesCS1A02G064800 in 306 worldwide wheat accessions. **Table S20.** The allele distribution of the SNP used in the RILs in the 306 worldwide wheat accessions. **Table S21.** The phenotypic data of RILs.

**Additional file 3.** Review history.

## Data Availability

The genotypes of 306 wheat accession used in this study have been deposited in the Genome Variation Map (https://bigd.big.ac.cn/gvm) under accession number GVM000463 [14, 54]. The sequence data of the 86 wheat accession used in this study were downloaded from the WheatUnion database (http://wheat.cau.edu.cn/WheatUnion/c_5/). The detailed information of all significantly associated SNP genotype data in Additional file 2: Table S3 belong to 306 wheat accession genotype, which can be obtained from GVM00463. The genotypic data of all the varieties in the first pedigree (Aimengniu and xiaoyan6) for Additional file 2: Tables S11 and S12 was downloaded from the WheatUnion database (http://wheat.cau.edu.cn/WheatUnion/c_5/). The raw phenotypic data for Additional file 2: Tables S8-S10 and S19 can be obtained from Additional file 2: Table S1. The raw phenotypic data for RIL can be obtained from the Additional file 2: Table S21. The genes in Additional file 2: Tables S5 [19, 55–78] and S6 [19, 56, 79–97] in the study were reported previously.
