# Supplementary material for: Genetic basis of geographical differentiation and breeding selection for wheat plant architecture traits
Source: Genome Biol. 2023 May 12;24:114. doi: 10.1186/s13059-023-02932-x (PMC10176713; doi:10.1186/s13059-023-02932-x)
Supplement: Supplementary file 2 — Additional file 2: Table S1. Summary of the 306 worldwide wheat accessions. Table S2. Comparison of the eight plant architecture traits between two environments. Table S3. Detailed information of all significantly associated SNPs for the investigated traits> 5). Table S4. The 330 identified loci associated with investigated traits in this study. Table S5. Overlap between the known genes/QTLs and 330 identified loci in this study. Table S6. XP-CLR scores for the known genes. Table S7. The haplotypes of four major loci for the length of the four internodes in 831 Chinese wheat accessions. Table S8. The phenotypic data for the varieties from the seven continents/regions. Table S9. The phenotypic data of the haplotypes of the four major loci for the length of the four internodes. Table S10. The distribution of haplotypes of the four internodes in 306 worldwide accessions. Table S11. The genotypic data of all the varieties in the first pedigree. Table S12. The genotypic data of all the varieties in the second pedigree. Table S13. The interaction between environments and haplotypes for the length of the four internodes. Table S14. The interaction between environments and haplotypes for the length of the four internodes. Table S15. The 432 wheat varieties used for the analysis of the evolutionary relationship of the haplotypes. Table S16. The haplotype combinations for the length of the four internodes in 306 worldwide wheat accessions. Table S17. The haplotype combinations for the length of the four internodes in 306 worldwide wheat accessions. Table S18. The distribution of haplotype combinations for the length of the four internodes in 831 Chinese wheat accessions. Table S19. The phenotypic data of the four major haplotypes of TraesCS1A02G064800 in 306 worldwide wheat accessions. Table S20. The allele distribution of the SNP used in the RILs in the 306 worldwide wheat accessions. Table S21. The phenotypic data of RILs. [file 13059_2023_2932_MOESM2_ESM.pdf]

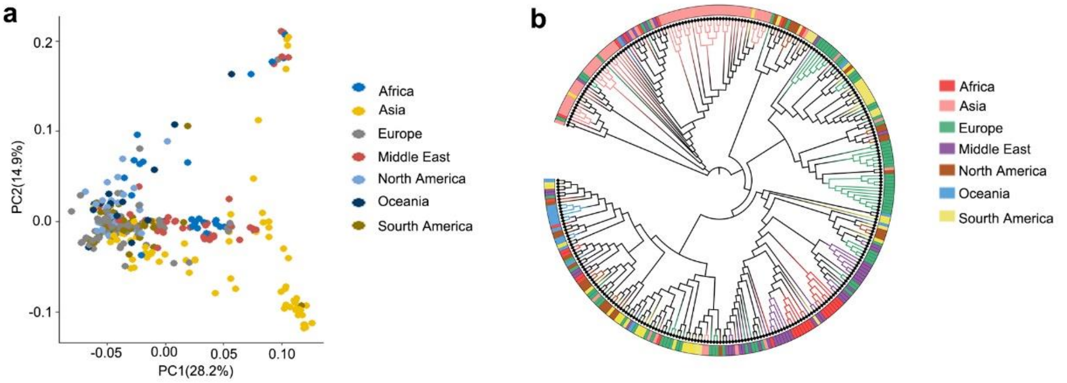

Figure S1. Phylogenetic relationships and population structures. a Principal components analysis (PCA) plots of the first two components (PC1 and PC2) of 306 worldwide wheat accessions. The colors of dots indicate the population and location**. b** Neighbor-joining (NJ) tree was built using a total of 40,710,923 SNPs. Branch colors reflect different populations. Accessions are arranged according to the populations and their geographic locations.

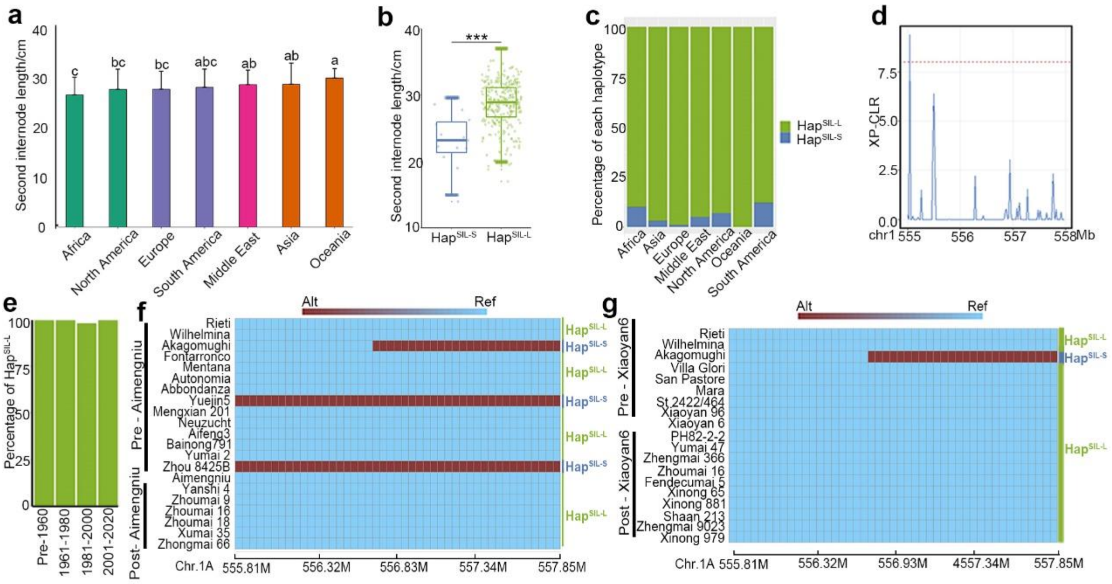

Figure S2. Geographical distribution and breeding selection of the haplotype blocks associated with the length of the second internode on chromosome 1A. a Length of the second internode across wheat accessions originating from the seven continents/regions. Data are shown as mean ± SD (n = 5). Significant differences were determined by ANOVA. Different lowercase letters indicate significant differences (P < 0.05). **b** Phenotypic distribution of the second internode length as a function of the two haplotypes on chromosome 1A. Data are means ± SD (n = 5). Significant differences were determined by a Student’s t-test (two sided, *** P < 0.001). **c** Proportion of accessions with each of the two haplotypes for each area. Asia means the region excluding the Middle East. **d** Comparison of XP-CLR score selection signals between Chinese landrace and cultivar for the major locus (Y1-SIL-1A-2, chr1A: 555809177-557861830) associated with second internode length. The red dotted line indicates the threshold (top 5% of scores). **e** Proportion of accessions harboring HapSIL-L in four times windows (pre-1960, 1961-1980, 1981-2000, 2001-2020) in the 831 wheat accessions released between 1900 and 2020. **f, g**) Distribution of haplotype blocks for the locus (Y1-SIL-1A-2) in two wheat pedigrees in Fig. 3f, h. Each row is a wheat accession and each column is a haplotype. Alleles that are identical to or different from that in the Chinese Spring genome are indicated by blue and red bars, respectively

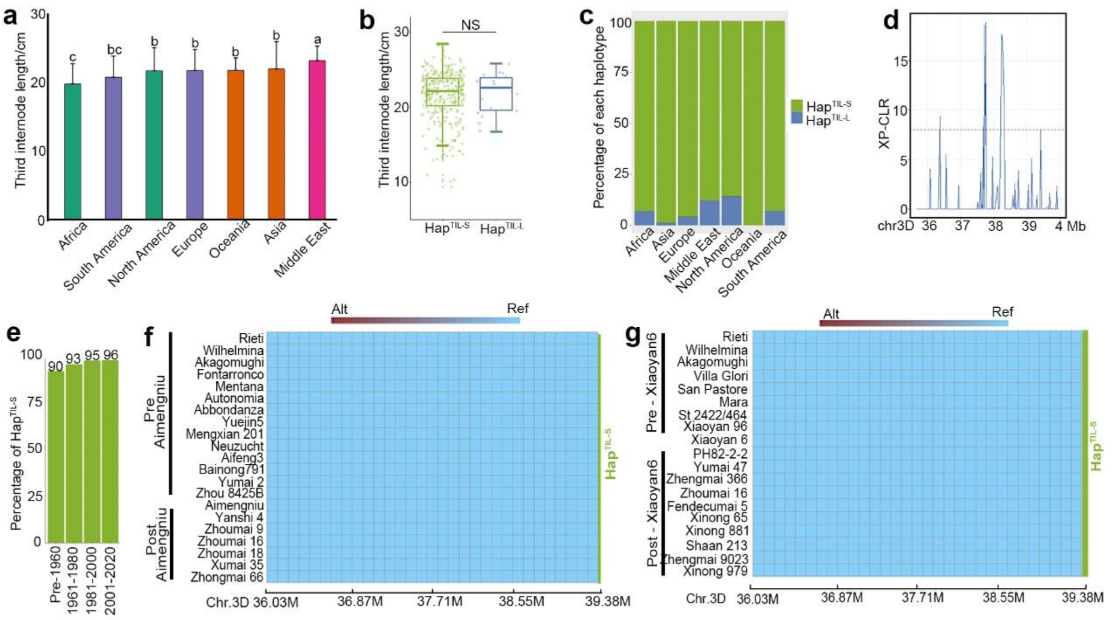

Figure S3. Geographical distribution and breeding selection of the haplotype blocks associated with the length of the third internode on chromosome 3D. a Length of the third internode across wheat accessions originating from the seven continents/regions. Data are means ± SD (n = 5). Significant differences were determined by ANOVA. Different lowercase letters indicate significant differences ($P < 0.05$). **b** Phenotypic distribution for third internode length as a function of the two haplotypes on chromosome 1A. Data are means ± SD (n = 5). Significant differences were determined by a Student's t-test (two sided, $**P < 0.01$). **c** Proportion of accessions with each of the two haplotypes for each area. Asia means the region excluding the Middle East. **d** Comparison of XP-CLR score selection signals between Chinese landraces and cultivars for the major locus (Y1-TIL-3D-1, chr3D:35,786,271-39,382,820) associated with the length of the third internode. The red dotted line indicates the threshold (top 5% of scores). **e** Proportion of accessions harboring the haplotype Hap^{TIL-S} in four times windows (pre-1960, 1961-1980, 1981-2000, 2001-2020) in the 831 wheat accessions released between 1900 and 2020. **f, g** Distribution of haplotype blocks of the locus (Y1-TIL-3D-1) in two wheat pedigrees in Fig. 3f, h. Each row is a wheat accession and each column is a haplotype. Alleles that are identical to or different from that in the Chinese Spring genome are indicated by blue and red bars, respectively.

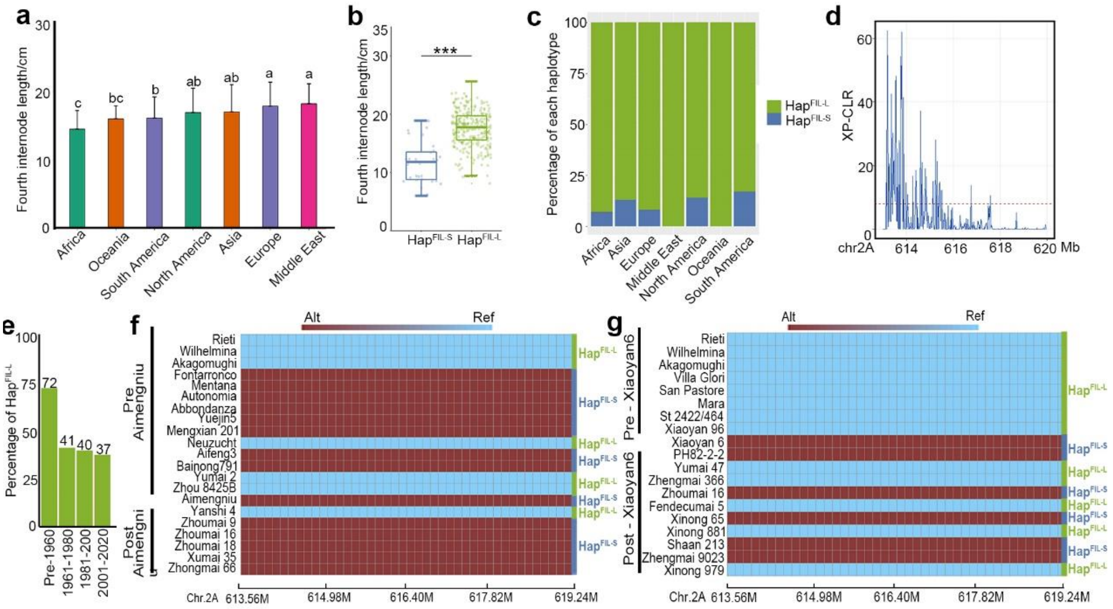

Figure S4. Geographical distribution and breeding selection of the haplotype blocks associated with the length of the fourth internode on chromosome 2A. a Length of the fourth internode across wheat accessions originating from the seven continents/regions. Data are means ± SD (n = 5). Significant differences were determined by ANOVA. Different lowercase letters indicate significant differences ($P < 0.05$). **b** Phenotypic distribution for the length of the fourth internode as a function of the two haplotypes on chromosome 1A. Data are means ± SD (n = 5). Significant differences were determined by a Student's t-test (two sided, *** $P < 0.001$). **c**Proportion of accessions with each of the two haplotypes for each area. Asia means the region excluding the Middle East. **d** Comparison of XP-CLR score selection signals between Chinese landraces and cultivars for the major locus (Y1-FIL-2A-3, chr2A:613,558,313-619,417,320) associated with the length of the fourth internode. The red dotted line indicates the threshold (top 5% of scores). **e** Proportion of accessions harboring the haplotype Hap^{FIL-L} in four times windows (pre-1960, 1961-1980, 1981-2000, 2001-2020) in the 831 wheat accessions released between 1900 and 2020. **f, g** Distribution of haplotype blocks of the locus (Y1-FIL-2A-3) in two wheat pedigrees in Fig. 3f, h. Each row is a wheat accession and each column is a haplotype. Alleles that are identical to or different from that in the Chinese Spring genome are indicated by blue and red bars, respectively.

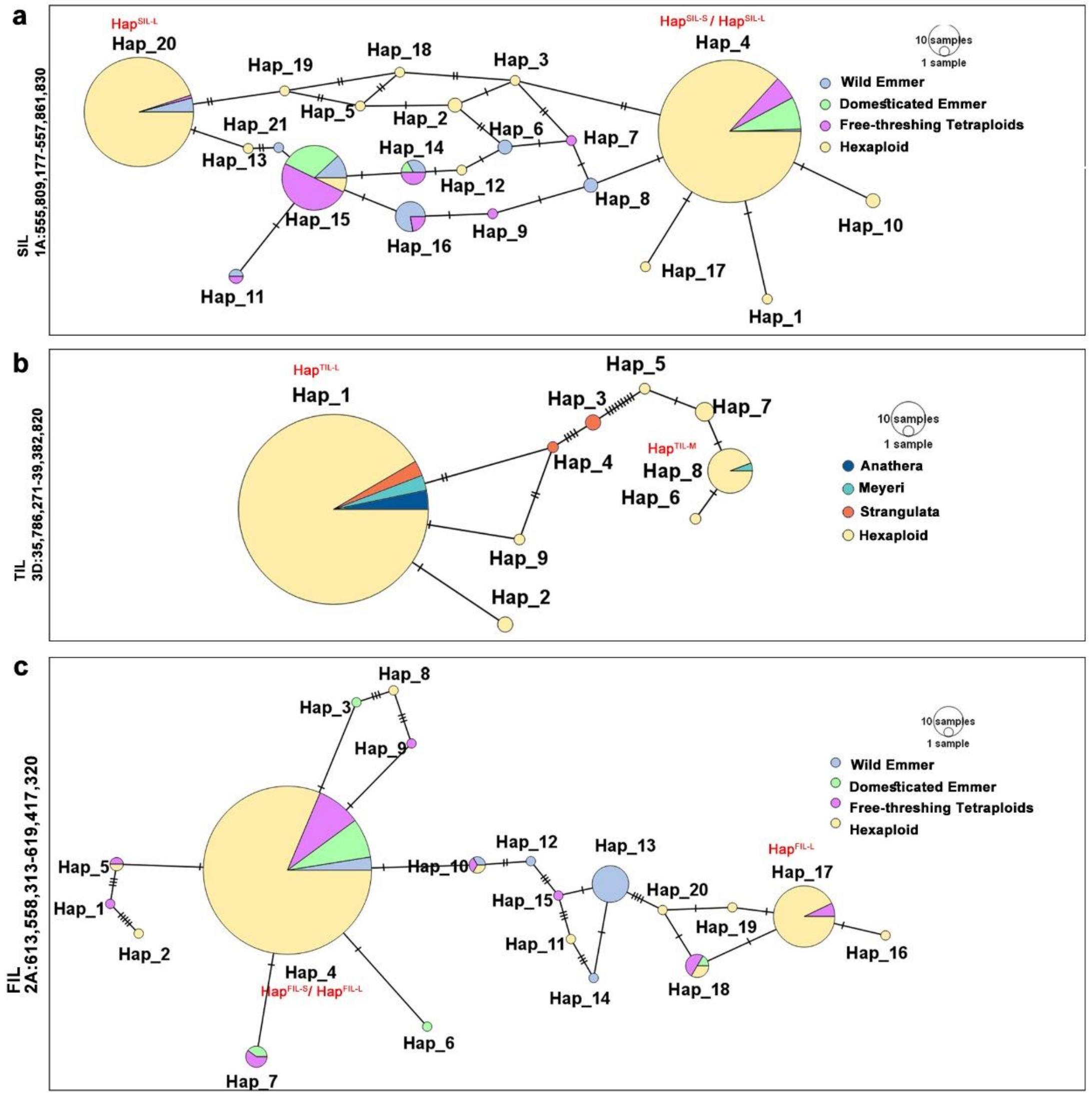

Figure S5. The evolutionary relationship of the haplotypes for the loci for the length of second internode (1A:555,809,177-557,861,830) (Figure S5a), third internode (3D:35,786,271-39,382,820) (Figure S5b), four internode (2A:613,558,313-619,417,320) (Figure S5c). The details were exhibited in additional file 2: Table S15
