# Supplementary material for: Genetic basis of geographical differentiation and breeding selection for wheat plant architecture traits
Source: Genome Biol. 2023 May 12;24:114. doi: 10.1186/s13059-023-02932-x (PMC10176713; doi:10.1186/s13059-023-02932-x)
Supplement: Supplementary file 3 — Additional file 3. Review history. [file 13059_2023_2932_MOESM3_ESM.docx]

Review History

**First round of review**

**Reviewer 1**

**Were you able to assess all statistics in the manuscript, including the appropriateness of statistical tests used?**

No, I do not feel adequately qualified to assess the statistics.

**Comments to author:**

In this manuscript, a GWAS was conducted using 306 historic worldwide collection of wheat cultivars/landraces to investigate variation in plant architecture traits by dissecting plant height components. They identified some genomic regions/haplotypes important to these components. Some of the genomic regions has pleiotropic effects on several component traits. Different internodes may be controlled by different genomic regions and plant height can be manipulated by reassembling these genomic regions to develop new cultivars with ideal plant height. The results are useful to wheat breeders worldwide. However, I still have several major concerns on experimental design, method description and content arrangement.

1. Detailed passport descriptions are missing for the wheat accessions used. How were the 306 accessions selected? Are they winter or spring type? Hard or soft wheat? Landraces or cultivars? These are important information for readers to understand the materials.

2. As described in the method, the experiment was done in two fields at one location. In each field, one plot (5 row) per accession was used (no replication). Also 5 plants were sampled a plot/accession in a field. No experimental design was mentioned. To my understand, the experiment can be considered as one location with two rep. However, plant height is an adaptation trait, it varies significantly with geographic locations as pointed by authors. For such large-scale experiment, at least another location with different eco-condition should be used in the study. In each location replications are required to ensure data repeatability.

3. It is not clear to me how the SNPs were imputed for 831 Chinese accessions. Were the 831 samples genotyped with some of the markers? If they were not genotyped with SNPs, based on what the haplotypes were imputed?

4. In result section, several places did not present the results, but discussion with many citations. However, in the discussion section, previously reported work on internode mapping were not discussed and compared with results found in this study. Are these genomic regions or QTLs identified in this study are novel or has been reported before? It sounds like this is the first study in internode QTL mapping. Also, did not cite other studies on these plant height traits in the discussion. Please move discussion part from the result section to discussion and add more discussion on related work reported by others.

5. Authors did comparison between three old Italian wheat cultivars and Chinese cultivars and found some regions in Chinese cultivars are different from these old Italian cultivars. However, I did not see any information on the genetic relationship of these Chinese cultivars with these Italian cultivars. Maybe I missed something. If they are not related in some way, we may not be able to do comparison between them.

**Reviewer 2**

**Were you able to assess all statistics in the manuscript, including the appropriateness of statistical tests used?**

Yes, and I have assessed the statistics in my report.

**Comments to author:**

The manuscript by Yangyang Liu et al., titled “Genetic basis of geographical differentiation and breeding selection for wheat plant architecture traits”, made an effort to dissect the genetic mechanisms and their geographical distribution differences underlying the plant architecture or plant node length of wheat based on GWAS. This is an interesting breeding related study.

However, the main limitation of the manuscript in Genome Biology context is that this is mainly focused on the summary of phenotypic and haplotype distribution differences during the breeding process, which should be published in breeding-research-oriented journals. A thorough exploration of the evolutionary relationship of the haplotypes, such as the gene introgression of different haplotypes, and interactions between various haplotypes and environments, is lacking in the article. Furthermore, QTL, which is a terminology conventionally used in QTL mapping/analysis based on the linkage of markers by crossing two or more parental strains, was used throughout the manuscript. It was redefined in the manuscript as “at least five nearby SNPs were above the significant threshold” in GWAS, which was based on the linkage disequilibrium and historical recombinants of the natural population. The associated SNP in GWAS is corresponding to the causal site of a gene rather than a QTL, and the phenomenon of clustered “nearby SNPs” is more likely derived from the unpruned dataset based on LD. The following are the detail comments throughout the manuscript (because the line numbers is not sequential, the comments were listed in sequential order):

1, In introduction, line 48. What is the ideotype or ideal plant architecture/node length of wheat?

2, In Results “Overview of plant architecture traits in this study”, lines 32-38. The length of the lowest (near the ground) node is always affected by the depth of soil covering. How did you control/normalized/unify it? Lines 45-60, why the correlation coefficients of main shoot length and shortest tiller length, and longest tiller length were so high (0.92 and 0.99), were they the same thing sometimes?

3, In results “Genome-wide association studies reveal shared and independent genetic determinants of plant architecture traits”, line 59. The threshold (-Log10(P-value) > 5.0) is not strict enough for 40,710,923 SNPs. Lines 1-4, the definition of QTL is weird. For the GWAS, is there any known gene picked as a control? How about the performance of other models such as MLM or CMLM? Lines 23-25, what is the genetic basis of the selection of 17 cultivar and 24 landraces from the association panel including 306 wheat accessions? What about the representativeness of the selected wheat lines.

4, In results “Genetic basis of geographical differentiation and breeding selection of internode

length”, lines 3-7, what’s the relationship between the 306 accessions in the association population and the 831 wheat accessions? Are they inclusion relationship, or overlapped?

5, In results “Peduncle length”, lines 40-43, what’s the relationship between 3 haplotype and the green revolution gene? The increasing percentage of the Hap^PL-M^ may result from the prevalence of the green revolution gene. Lines 51-10 (next page), this part is confusing. What about the genotypes of the two founders? Is there any other founder involved in the breeding process? What’s the relationship between the “major global genotypes (Rieti, Whlhelmina, and Akagomughi)” and the haplotypes analyzed in this section?

6, In results “Length of the fourth internode”, lines 43-44. If there is a possible that other founders involved, it can’t be excluded the possible that other haplotypes/genotypes used in the article were introduced from other founders besides the two founders mentioned in this manuscript.

**Authors Response**

**Point-by-point responses to the reviewers’ comments:**

Reviewer #1:

In this manuscript, a GWAS was conducted using 306 historic worldwide collection of wheat cultivars/landraces to investigate variation in plant architecture traits by dissecting plant height components. They identified some genomic regions/haplotypes important to these components. Some of the genomic regions has pleiotropic effects on several component traits. Different internodes may be controlled by different genomic regions and plant height can be manipulated by reassembling these genomic regions to develop new cultivars with ideal plant height. The results are useful to wheat breeders worldwide. However, I still have several major concerns on experimental design, method description and content arrangement.

1. Detailed passport descriptions are missing for the wheat accessions used. How were the 306 accessions selected? Are they winter or spring type? Hard or soft wheat? Landraces or cultivars? These are important information for readers to understand the materials.

*Authors’ response 1: Thanks for the kind suggestion. The multiple originations of wheat domestication have led to genetic and phenotypic variation in different regions worldwide. The 306 accessions were selected based on the genetic and phenotypic diversity across worldwide wheat accessions. The 306 accessions included 96 winter varieties and 178 spring varieties, 175 hard varieties and 96 soft varieties, 179 landraces and 38 traditional cultivars (Additional file 1: Table S1). According to the kind suggestion, we included these important information in Additional file 1: Table S1. In addition, we added this important information in the manuscript.*

*The modifications in the manuscript (line 112-116 in the revised manuscript with track changes): “The multiple originations of wheat domestication have led to genetic and phenotypic variation in different regions worldwide. The 306 accessions were selected based on the genetic and phenotypic diversity across worldwide wheat accessions. The 306 accessions included 96 winter varieties and 178 spring varieties, 175 hard varieties and 96 soft varieties, 179 landraces and 38 traditional cultivars (Additional file 1: Table S1)”*

2. As described in the method, the experiment was done in two fields at one location. In each field, one plot (5 row) per accession was used (no replication). Also 5 plants were sampled a plot/accession in a field. No experimental design was mentioned. To my understand, the experiment can be considered as one location with two rep. However, plant height is an adaptation trait, it varies significantly with geographic locations as pointed by authors. For such large-scale experiment, at least another location with different eco-condition should be used in the study. In each location replications are required to ensure data repeatability.

*Authors’ response 2: Thanks for the kind suggestion. We included more data which obtained in 2022 and reanalyzed all the data to ensure data repeatability according to the suggestions (Additional file 1: Table S2). In this case, we have 5 plants for one plot/replicate, two plots/replicates for each year, and phenotypic data from two years. In addition, we reanalyzed the data and found the consistent results between the two years.*

*The modifications in the manuscript (line 716-720 in the revised manuscript with track changes, Additional file 1: Table S2, 4, 12): “Seeds for 306 worldwide wheat accessions were sown with two replicates to ensure data repeatability in Zhaoxian, Hebei province, China (37o27’ N, 113o30’ E, altitude 78 m) in 2020-2021 and 2021-2022. At For each replicate, six rows were planted per accession, with each row (1.5 m in length) including 15 plants, with 10 cm between rows”*

3. It is not clear to me how the SNPs were imputed for 831 Chinese accessions. Were the 831 samples genotyped with some of the markers? If they were not genotyped with SNPs, based on what the haplotypes were imputed?

*Authors’ response 3: For the 831 Chinese accessions, we genotyped all the accessions with wheat 660K SNP array according to previous work (reference: The Wheat 660K SNP array demonstrates great potential for marker assisted selection in polyploid wheat). We clarified the information in the revised manuscript.*

*The modifications in the manuscript (line 756-757 in the revised manuscript with track changes): “For the 831 Chinese accessions, we genotyped all the accessions with wheat 660K SNP array [54]”*

4. In result section, several places did not present the results, but discussion with many citations. However, in the discussion section, previously reported work on internode mapping were not discussed and compared with results found in this study. Are these genomic regions or QTLs identified in this study are novel or has been reported before? It sounds like this is the first study in internode QTL mapping. Also, did not cite other studies on these plant height traits in the discussion. Please move discussion part from the result section to discussion and add more discussion on related work reported by others.

*Authors’ response 4: (1) Thanks for the kind suggestion. We added the reported work on internode mapping and compared with results found in this study (line 622-630 in the revised manuscript with track changes). To the best of our knowledge, in wheat, the only publication reported the QTLs of the internode length using two biparental populations without the physical positions in wheat genome, since the information of reference genome was not available at that time. We cannot compared with identified QTLs with our work because of the lack of physical positions. Instead, we compared our identified loci with known QTLs/genes identified by other agronomic traits.*

*The modifications in the manuscript (line 622-630, 648-671 in the revised manuscript with track changes): “To the best of our knowledge, in wheat, some genetic work related to peduncle have been reported [26, 27]. However, few genetic studies have specifically focused on dissected the length of internodes or on the length of the shortest and longest tillers within individual plants. The only publication reported the QTLs of the internode length using two biparental populations without the physical positions in wheat genome, since the information of reference genome was not available at that time [28]. Some identified loci in this study were overlapped with reported QTLs or genes in previous work. Nevertheless, a big proportion of the identified loci, especially these loci associated with four internode, are novel relative to previous studies.”*

*“Based on the GWAS results in this study, we identified Rht-D1 as a candidate gene for the length of the second internode, the third internode, the main shoot, the shortest tiller and the longest tiller (Fig. 2b, Additional file 1: Table S35). Plant height has been reported multiple times to influence thousand grain weight (TGW) and grain number [18, 29, 30]. In agreement with this notion, GWAS results for peduncle length, the length of the main shoot, the shortest tiller and the longest tiller obtained in this study detected several QTLs that overlap with previous genomic regions reported to be linked to TGW and grain number (Fig. 2b, Additional file 1: Table S35): QKNS-2A-AN [31], QTKW-2A-AN [32], and QTKW.ndsu.7D [33]. In wheat, down regulated expression of PIN-FORMED1 (TaPIN1) via RNA interference decreased TGW and grain number [34]. Here, we identified TaPIN1-6D as a candidate gene for the length of the main shoot and the longest tiller (Fig. 2, Additional file 1: Table S35). Photoperiod-1 (Ppd-1) has major effects on the adaptability of bread wheat to local environments by regulating flowering time [35, 36]. Ppd-D1 has also been shown to be associated with plant height, grain number, and TGW [37, 38]. Our results suggested that Ppd-D1 may also regulate the length of the main shoot, the longest tiller, the shorted tiller, the peduncle, the third internode, the fourth internode, and the length difference between the shortest and the longest tiller in wheat (Fig. 2, Additional file 1: Table S35). Mature fruits from apple (Malus × domestica) with antisense suppression of SEPALLATA1/2 (SEP1/2) transcripts have lower ethylene levels, higher auxin contents, and reduced transcription of ripening-related genes compared to control apples [39, 40]. OsMADS1 determines grain size and floret development in rice (Oryza sativa) [41, 42]. Wheat LEAFY HULL STERILE1 (WLHS1) is a homolog of OsMADS1 [43]. Both SEP1/2-4A and WLHS1-4A are candidate genes for the length of the longest tiller in this study”*

*(2) We removed the citation of these plant height traits in the discussion.*

*The modifications in the manuscript (line 648-671 in the revised manuscript with track changes).*

*(3) We moved discussion part from the result section to discussion and added more discussion on related work reported by others.*

*The modifications in the manuscript (line 189-195, 618-630 in the revised manuscript with track changes): “We took TEOSINTE BRANCHED1 (TB1) as a control, because recent work reported that TB1 regulates height and stem internode length in bread wheat [15]. The genomic region (Chr4D: 15938883-19054796) including TB1 was associated with the length of second internode, third internode, shortest tiller, longest tiller and main shoot (Additional file 1: Table S5), suggesting its potential connection with internode length, which is consistent with previous finding [15] (Line 189-195)”*

5. Authors did comparison between three old Italian wheat cultivars and Chinese cultivars and found some regions in Chinese cultivars are different from these old Italian cultivars. However, I did not see any information on the genetic relationship of these Chinese cultivars with these Italian cultivars. Maybe I missed something. If they are not related in some way, we may not be able to do comparison between them.

*Authors’ response 5: Thanks for the kind suggestion. The old Italian cultivars made great contributions to these Chinese cultivars. In other words, these Chinese cultivars were developed by crossing the old Italian cultivars with other varieties. According to the kind suggestion, we included more information in the manuscript. The detailed connections were also displayed in our previous work (Reference: Resequencing of 145 Landmark Cultivars Reveals Asymmetric Sub-genome Selection and Strong Founder Genotype Effects on Wheat Breeding in China). In addition, the genetic relationship of these Chinese cultivars with these Italian cultivars were revealed in our previous work (Reference: Resequencing of 145 Landmark Cultivars Reveals Asymmetric Sub-genome Selection and Strong Founder Genotype Effects on Wheat Breeding in China).*

*The modifications in the manuscript (line 294-318 in the revised manuscript with track changes, Additional file 1: Table S10, 11): “To confirm the changes in frequency for the haplotypes at Y1-PL-1A-1, we used two important pedigrees in Chinese wheat breeding. We examined the breeding histories of two important cultivars (Aimengniu, Xiaoyan6) that were initially released in the early 1980s. Our collection included a variety of pedigree contributors and subsequently released derived cultivars for both Aimengniu and Xiaoyan6 founder genotypes (Fig. 3f,h). The genetic contribution of founder genotypes to their derived cultivars was reported in our previous work [9]. We examined the distribution of three haplotypes (HapPL-S, HapPL-M, and HapPL-L) in the two pedigrees of two founder genotypes (Aimengniu, Xiaoyan6) (Fig. 3f, h). Both pedigrees were ultimately derived from three major global genotypes (Rieti, Wilhelmina, and Akagomughi) between the 1920s and 1970s [9]. In the first pedigree, three founder genotypes (Rieti, Wilhelmina, and Akagomughi) contributed to derived cultivars: Mentana, Autonomia, Abbondanza, Mengxian 201, Aimengniu. More recently, Zhoumai 9, Zhoumai 16, Xumai 35, Zhoumai 18, Zhongmai 66 derived from Aimengniu (Fig. 3f). In addition, some other varieties (e.g. Fontarronco, Yuejin5, Neuzucht, Aifeng5, Bainong791, Yanshi4, Zhou8425B) contributed to derived cultivars in the first pedigree. In the second pedigree, Villa Glori, San Pastore, St 2422/464 and Xiaoyan6 were the derivatives of the same founder genotypes (Rieti, Wilhelmina, and Akagomughi). Next, Xinong 881, Zhengmai 9023, Zhengmai 366, Xinong 979 and Fengdecunmai 5 derived from Xiaoyan6 (Fig. 3h). Moreover, some other varieties (e.g. Mara, Xiaoyan96, Xinong65, Yumai47, Shaan 213, Zhengmai16) contributed to derived cultivars in the second pedigree. All the SNP information of the varieties in the two pedigrees can be obtained from our previous work [9].”*

Reviewer #2:

The manuscript by Yangyang Liu et al., titled “Genetic basis of geographical differentiation and breeding selection for wheat plant architecture traits”, made an effort to dissect the genetic mechanisms and their geographical distribution differences underlying the plant architecture or plant node length of wheat based on GWAS. This is an interesting breeding related study.

However, the main limitation of the manuscript in Genome Biology context is that this is mainly focused on the summary of phenotypic and haplotype distribution differences during the breeding process, which should be published in breeding-research-oriented journals. A thorough exploration of the evolutionary relationship of the haplotypes, such as the gene introgression of different haplotypes, and interactions between various haplotypes and environments, is lacking in the article. Furthermore, QTL, which is a terminology conventionally used in QTL mapping/analysis based on the linkage of markers by crossing two or more parental strains, was used throughout the manuscript. It was redefined in the manuscript as “at least five nearby SNPs were above the significant threshold” in GWAS, which was based on the linkage disequilibrium and historical recombinants of the natural population. The associated SNP in GWAS is corresponding to the causal site of a gene rather than a QTL, and the phenomenon of clustered “nearby SNPs” is more likely derived from the unpruned dataset based on LD. The following are the detail comments throughout the manuscript (because the line numbers is not sequential, the comments were listed in sequential order):

*Authors’ response: (1) According to the suggestion, we displayed the evolutionary relationship of the haplotypes in diploid, tetraploid and hexaploid wheat. (To be honest, we did not completely understand this question: the evolutionary relationship of the haplotypes, such as the gene introgression of different haplotypes. If we did not answer it properly, we will be very happy to response to this suggestion after the reviewer’s further explanation).*

*The modifications in the manuscript (line 445-473 in the revised manuscript with track changes, Fig. 4): “To evaluate the evolutionary relationship of the haplotypes for loci of the length of the four internode, in addition to the 306 hexaploid wheat accessions, we used 126 additional accessions including 30 Aegilops tauschii, 96 tetraploid wheat varieties, which have been sequenced previously [7]. We extracted SNPs that are in high linkage disequilibrium in a haplotype and used popArt [16] to visualize the network (Fig. 4a).*

*The haplotypes of loci for peduncle length (PL) locus (1A:45,791,400-49,094,709), second internode length (SIL, 1A:555,809,177-557,861,830) and fourth internode length (FIL, 2A:613,558,313-619,417,320) appeared to derive from wild emmer. In addition, the introgression of these haplotypes from domesticated emmer and free-threshing tetraploids may partly contribute to their appearance in hexaploid wheat. This evolution process of these haplotypes for these two loci is consistent with the evolutionary history of hexaploid wheat. The haplotypes of locus for third internode length (TIL, 3D:35,786,271-39,382,820) may infiltrated from Anathera, Meyeri, Strangulata. Consistently, our previous work indicated that Strangulata is the donor of D genome [7].*

*The haplotype of PL locus (1A:45,791,400-49,094,709) in Mengxian201 was unclear in the first pedigree (Fig. 3g). We therefore examined the potential genetic donor of haplotypes for Mengxian201. The potential donors are four free-threshing tetraploids and one domesticated emmer indicated by fd > 0.9 (Fig. 4b). To further seek the donor of Mengxian201, we analysized the haplotype blocks on this locus with its possible donors, one free-threshing tetraploids B016 turns out to be the most possible donor of Mengxian201, because it has the most similar haplotype blocks with Mengxian201 among all five candidates and fd values is nearly equal to 1 across the locus (Fig. 4c, d). Furthermore, the minimum IBS distance also support this hypothesis, the value of IBS distance between Mengxian201 and B016 is even smaller than that with the hexaploidy landrace Aifeng3 (Fig. 4c). Our work indicated that free-threshing tetraploids B016 is the most possible donor of Mengxian201, however, it might not be the direct donor, since we cannot exclude the other possibilities during wheat breeding, such as natural outcrossing, heterozygous parent.”*

*(2) We evaluated the interaction between haplotypes and environemnts and observed obvious GXE effects on the phenotypes.*

*The modifications in the manuscript (line 474-483 in the revised manuscript with track changes): “We observed obvious differences of the phenotypic values between the two environments for the length of four internodes (Additional file 1: Table S2). Notably, the genotypic values for the length of the first and second internodes were higher in 2021 than those in 2022. However, the genotypic values for the length of the third and fourth internodes were higher in 2022 than those in 2021. The Shukla model [17]was used to evaluate the interaction between genotypes and environments (Additional file 1: Table S12). Obvious interactions between haplotypes and environments for four haplotypes (HapPL-S, HapSIL-L, HapSIL-S, HapTIL-L) were observed in this study (Additional file 1: Table S12). In addition, the results suggested the relative stability of other haplotypes of the length of four internodes (Additional file 1: Table S12).”*

*(3) Thanks for the professional suggestion, we defined the associated genomic regions as locus instead of QTLs.*

*The modifications in the manuscript (line 156-169 in the revised manuscript with track changes): “We used linkage disequilibrium (LD) and connections between markers to delineate about 330 significantly associated loci, when at least five nearby SNPs were above the significance threshold, associated with the eight plant architecture traits (Additional file 1: Table S4).”*

1, In introduction, line 48. What is the ideotype or ideal plant architecture/node length of wheat?

*Authors’ response 1: According to Donald (1968), a crop ideotype is a weak competitor and make a minimum demand on resources per unit of dry matter produced. An ideal plant architecture is of high efficiency relative to its environmental resources. Therefore, ideal plant architecture/node length of wheat is different in variable environments (e.g. drought, lodging).*

*The modifications in the manuscript (line 66-70 in the revised manuscript with track changes): “According to Donald [3], a crop ideotype is a weak competitor and make a minimum demand on resources per unit of dry matter produced. An ideal plant architecture is of high efficiency relative to its environmental resources. Ideal plant architecture/node length of wheat is different in variable environments (e.g. drought, lodging).”*

2, In Results “Overview of plant architecture traits in this study”, lines 32-38. The length of the lowest (near the ground) node is always affected by the depth of soil covering. How did you control/normalized/unify it? Lines 45-60, why the correlation coefficients of main shoot length and shortest tiller length, and longest tiller length were so high (0.92 and 0.99), were they the same thing sometimes?

*Authors’ response 2: Thanks for the question. For the sowing, we used a self-made hole punch to ensure the consistency of plant spacing and sowing depth. The punch is welded on a 1-meter-long steel plate, on which steel columns with equal spacing are evenly distributed. These steel columns are thick at the top and thin at the bottom, and have equal length, which allowed us to ensure that the hole depth is consistent.*

*The modifications in the manuscript (line 720-724 in the revised manuscript with track changes): “For the sowing, we used a self-made hole punch to ensure the consistency of plant spacing and sowing depth. The punch is welded on a 1-meter-long steel plate, on which steel columns with equal spacing are evenly distributed. These steel columns are thick at the top and thin at the bottom, and have equal length, which allowed us to ensure that the hole depth is consistent.”*

3, In results “Genome-wide association studies reveal shared and independent genetic determinants of plant architecture traits”, line 59. The threshold (-Log10(P-value) > 5.0) is not strict enough for 40,710,923 SNPs. Lines 1-4, the definition of QTL is weird. For the GWAS, is there any known gene picked as a control? How about the performance of other models such as MLM or CMLM? Lines 23-25, what is the genetic basis of the selection of 17 cultivar and 24 landraces from the association panel including 306 wheat accessions? What about the representativeness of the selected wheat lines.

*Authors’ response 3: (1) We first removed the linked SNPs, obtained 2756291 SNPs, and further determined the P value as 1/2756291=3.63E-7. According the GWAS results, we found that the threshold (-Log10 (3.63E-7) = 6.44 may induce the loss of some important peaks. Therefore, we used significant P-value thresholds (P <10–5 ) were set to control genome-wide type I errors according to the previous study that included identical SNP number in wheat. (reference: Resequencing of 145 Landmark Cultivars Reveals Asymmetric Sub-genome Selection and Strong Founder Genotype Effects on Wheat Breeding in China).*

*The modifications in the manuscript (line 781-783 in the revised manuscript with track changes): “We used significant P-value thresholds (P <10–5 ) to control genome-wide type I errors according to previous study that included identical SNP number in wheat [9].”*

*(2) I am sorry we did not describe it clearly. In this study, we used mixed linear model (MLM). We clarified it according to the suggestion.*

*The modifications in the manuscript (line 773-776 in the revised manuscript with track changes): “Therefore, the GWAS was conducted using GEMMA (version 0.98.4) by fitting the mixed linear model (MLM) association expedited (EMMAX) algorithm, including kinship as a correlation matrix.”*

*(3) We defined the genomic region as locus instead of QTL according to the suggestion.*

*The modifications in the manuscript (line 165-169 in the revised manuscript with track changes): “We used linkage disequilibrium (LD) and connections between markers to delineate about 330 significantly associated loci, when at least five nearby SNPs were above the significance threshold, associated with the eight plant architecture traits (Additional file 1: Table S4).”*

*(4) We took TEOSINTE BRANCHED1 (TB1) as a control, because recent work reported that TB1 regulates height and stem internode length in bread wheat (reference: TEOSINTE BRANCHED1 regulates height and stem internode length in bread wheat).*

*The modifications in the manuscript (line 189-195 in the revised manuscript with track changes): “We took TEOSINTE BRANCHED1 (TB1) as a control, because recent work reported that TB1 regulates height and stem internode length in bread wheat [15]. The genomic region (Chr4D:18463838-18472387) including TB1 was associated with the length of second internode, third internode, shortest tiller, longest tiller and main shoot (Additional file 1: Table S5), suggesting its potential connection with internode length, which is consistent with previous finding [15].”*

*(5) These 59 Chinese accessions (17 cultivar and 42 landraces) were selected to display the genomic selection during Chinese wheat breeding process, which will be further used to examine the selection of the associated peaks identified by GWAS. The 59 Chinese accessions are important varieties during Chinese wheat breeding process.*

*The modifications in the manuscript (line 217-223 in the revised manuscript with track changes): “To confirm the selected loci in the wheat genome during the gradual improvement of grain yield in China, we selected 59 Chinese wheat accessions (17 cultivars versus 42 landraces) from the 306 wheat accessions for further analysis. These 59 Chinese accessions were selected to display the genomic selection during Chinese wheat breeding process, which will be further used to examine the selection of the associated peaks identified by GWAS. The 59 Chinese accessions are important varieties during Chinese wheat breeding process.”*

4, In results “Genetic basis of geographical differentiation and breeding selection of internode

length”, lines 3-7, what’s the relationship between the 306 accessions in the association population and the 831 wheat accessions? Are they inclusion relationship, or overlapped?

*Authors’ response 4: Thanks for the kind questions. The 306 accessions are landraces and traditional cultivars, most of the 831 Chinese accessions are modern cultivars. The 831 wheat accessions were used to display the direction of the changes of haplotype composition were introduced from other countries or developed in Chinese wheat breeding. Between 306 worldwide accessions and 831 Chinese accessions, there are only seven overlapped accessions. We clarified the information in the revised manuscript.*

*The modifications in the manuscript (line 254-258 in the revised manuscript with track changes):“In addition, we characterized the extent and direction of the changes of haplotype composition in the 831 wheat accessions (most of the 831 Chinese accessions are modern cultivars) that were introduced from other countries or developed in China since 1900 (Additional file 1: Table S7).”*

5, In results “Peduncle length”, lines 40-43, what’s the relationship between 3 haplotype and the green revolution gene? The increasing percentage of the HapPL-M may result from the prevalence of the green revolution gene. Lines 51-10 (next page), this part is confusing. What about the genotypes of the two founders? Is there any other founder involved in the breeding process? What’s the relationship between the “major global genotypes (Rieti, Whlhelmina, and Akagomughi)” and the haplotypes analyzed in this section?

*Authors’ response 5: (1) To the best of our knowledge, we did not detect the connection between the haplotypes of peduncle length and green revolution gene. As we mentioned in the manuscript, TraesCS1A02G064800 is a candidate gene for this locus of peduncle length.*

*The modifications in the manuscript (line 563-565 in the revised manuscript with track changes): “Therefore, we identified TraesCS1A02G064800 as a candidate gene that controlled peduncle length (Fig. 6b). The orthologue of TraesCS1A02G064800 in Arabidopsis is Trehalose-6-P synthase 1.”*

*(2) We modified this part (line 51-10 next page) according to the suggestion.*

*The modifications in the manuscript (line 294-300 in the revised manuscript with track changes): “To confirm the changes in frequency for the haplotypes at Y1-PL-1A-1, we used two important pedigrees in Chinese wheat breeding. We examined the breeding histories of two important cultivars (Aimengniu, Xiaoyan6) that were initially released in the early 1980s. Our collection included a variety of pedigree contributors and subsequently released derived cultivars for both Aimengniu and Xiaoyan6 founder genotypes (Fig. 3f,h). The genetic contribution of founder genotypes to their derived cultivars was reported in our previous work [9].”*

*(3) We added the SNP information of the varieties in the two pedigrees, including the two founders in Additional file 1: Table S10, 11 according to the suggestion. Meanwhile, the information can be obtained from our previous work (Reference: Resequencing of 145 Landmark Cultivars Reveals Asymmetric Sub-genome Selection and Strong Founder Genotype Effects on Wheat Breeding in China). The major global genotypes displayed different haplotypes of the peak analyzed in this section.*

6, In results “Length of the fourth internode”, lines 43-44. If there is a possible that other founders involved, it can’t be excluded the possible that other haplotypes/genotypes used in the article were introduced from other founders besides the two founders mentioned in this manuscript.

*Authors’ response 6: Thank you very much for the professional suggestion. According to the suggestion, we included other founders and detected that the haplotypes were introduced from other founders besides the two founders mentioned in this manuscript.*

*The modifications in the manuscript (line 310-318, 419-432 in the revised manuscript with track changes): “In addition, some other varieties (e.g. Fontarronco, Yuejin5, Neuzucht, Aifeng5, Bainong791, Yanshi4, Zhou8425B) contributed to derived cultivars in the first pedigree.In the second pedigree, Villa Glori, San Pastore, St 2422/464 and Xiaoyan6 were the derivatives of the same founder genotypes (Rieti, Wilhelmina, and Akagomughi). Next, Xinong 881, Zhengmai 9023, Zhengmai 366, Xinong 979 and Fengdecunmai 5 derived from Xiaoyan6 (Fig. 3h). Moreover, some other varieties (e.g. Mara, Xiaoyan96, Xinong65, Yumai47, Shaan 213, Zhengmai16) contributed to derived cultivars in the second pedigree. All the SNP information of the varieties in the two pedigrees can be obtained from our previous work [9].”*

*“Y1-FIL-2A-3 and B-FIL-2A-1 were clearly selected between Chinese landraces and cultivars (Additional file 1: Table S4; Additional file 2: Figure S3d). The two haplotypes at Y1-FIL-2A-3 displayed changes in their frequencies across breeding periods. In the 831 wheat accessions, HapFIL-L was the major haplotype in 72% of accessions released prior to 1960, this percentage decreased from 72% before1960 to 37% after 2000 (Additional file 1: Table S7; Additional file 2: Figure S3e ). In the two pedigrees, all the three founder genotypes (Rieti, Wilhelmina, and Akagomughi) harbored HapFIL-L. In the first pedigree, most derived cultivars carried HapFIL-S, four derived cultivars (i.e. Neuzucht, Yumai2, Zhou8425B, Yanshi4) had HapFIL-L (Additional file 1: Table S10; Additional file 2: Figure S3f).In the second pedigree, Xiaoyan6, PH62-2-2, Zhoumai16, Xinong65, Shaan 213, Zhoumai9023 and Xinong979 had HapFIL-S (Additional file 1: Table S11; Additional file 2: Figure S3g).”*

**Second round of review**

**Reviewer 2**

The authors have provided elaborate explorations and additional information regarding my main recommendation to investigate the evolutionary relationships among the wheat germplasms rather than merely focusing on the humdrum phenotype and haplotype distributions. I basically approve of the responses and supplementary contents. There are still some flaws need to be minor revised:

1. Line 86, “…study (GWAS)…” to “…studies (GWAS)…”.

2. Line 88, “…GWAS has” to “…GWAS have…”.

3. In Figure 1c, what did the trees on the edge of the plot represent? They are not mentioned in the manuscript. I don’t think it is useful for the conclusion even though I know they were generated from the R package by default. If yes, please explain them, if not, just remove them, otherwise it is confused.

4. In lines 161-162, the sentence “we performed GWAS for two environments…” is confusing. Did you use the two environmental factors as traits to perform GWAS? Or what you would like to say was that we performed GWAS for the BLUE values of eight plant architecture traits IN two environments?

5. Line 168, “associated with the eight plant architecture traits” to “associated with at least one of the eight plant architecture traits”.

6. I suggest placing the sentences “We took *TEOSINTE BRANCHED1*…, which is consistent with the previous finding.” in front of “Notably, the major loci for the length of main shoot …”.

7. Please add more details to the Figure legends, especially for the Figure 4, so that readers can comprehend the findings simply from glancing at the Figures. “PL” to “Peduncle length (PL)” in line 436.

8. Line 457, adjust the font size of “may”.

9. In the first two paragraphs of the “Manipulating main shoot length through peduncle” section, it is confused that 5,768 SNPs were identified as significantly associating with PL. Is it the truth? Or 5,768 SNPs were used to conduct the GWAS? If the original is right, why only four SNPs were studied furtherly? What about the remaining 5,764 SNPs?

10. Line 564, the citation of “(Fig. 6b)” is not appropriate. Fig. 6b is just a LD block plot. I can’t get any gene information from it. And please label the SNPs so that the corresponding LD block can be recognized, such as connecting the target SNPs with the corresponding LD blocks using a line.

11. Figure 6f is not quoted in the manuscript. And the “Fig. 5g” in lines 591 and 593 incorrectly quoted.

12. In “Identification of putative selective sweeps” part, please clarify what software was used for the XP-CLR calculation and which population was used as the reference population.

13. Line 852, “…because the fd statistic value…” to “…because the negative fd statistic value…”

14. Use italic format for the R package names.

After going through the manuscript, remarks, and responses, I think the authors addressed the concerns from the reviewee #1 generally, except for the first question from reviewee #1:

1. "The multiple originations of wheat domestication have led to genetic and phenotypic variation in different regions worldwide. The 306 accessions were selected based on the genetic and phenotypic diversity across worldwide wheat accessions."

Performing population structure analyses, such as phylogenetic tree, or PCA, would be helpful to clarify the genetic diversity of the 306 accession rather than to prove the genetic and phenotypic variation by emphasizing the multiple originations and worldwide collection, which makes less sense for the question because even though they were from different geographic zones, they might underwent same selection bottleneck which led to the possible of convergent evolution.

2. "175 hard varieties and 96 soft varieties", which might be confusing for readers who are not studying wheats: hard and soft for what, seed?

3. " The 306 accessions included 96 winter varieties and 178 spring varieties, 175 hard varieties and 96 soft varieties, 179 landraces and 38 traditional cultivars". I don't think this statement is clear. It is easy to be misunderstood as 306=95+178+175+96+179+38. Winter/spring, soft/hard, and landrace/traditional cultivars are 3 inclusive categories. If the MS emphasized the domestication, it might be just using "The 306 accessions included 179 landraces and 38 traditional cultivars", the remaining information (hard/soft, winter/spring) can be referred to Table S1. Or any better expression patter.

**Authors Response**

**Point-by-point responses to the reviewers’ comments:**

Reviewer #2:

The authors have provided elaborate explorations and additional information regarding my main recommendation to investigate the evolutionary relationships among the wheat germplasms rather than merely focusing on the humdrum phenotype and haplotype distributions. I basically approve of the responses and supplementary contents. There are still some flaws need to be minor revised:

1. Line 86, “…study (GWAS)…” to “…studies (GWAS)…”.

*Authors’ response 1: Thanks. We modified it accordingly (line 85 in the revised manuscript with track changes).*

2. Line 88, “…GWAS has” to “…GWAS have…”.

*Authors’ response 2: Thanks. We modified it accordingly (line 87 in the revised manuscript with track changes).*

3. In Figure 1c, what did the trees on the edge of the plot represent? They are not mentioned in the manuscript. I don’t think it is useful for the conclusion even though I know they were generated from the R package by default. If yes, please explain them, if not, just remove them, otherwise it is confused.

*Authors’ response 3: Thanks. We removed the trees in the Fig. 1c according to the suggestion.*

4. In lines 161-162, the sentence “we performed GWAS for two environments…” is confusing. Did you use the two environmental factors as traits to perform GWAS? Or what you would like to say was that we performed GWAS for the BLUE values of eight plant architecture traits IN two environments?

*Authors’ response 4: Thanks. We modified it accordingly.*

*The modifications in the manuscript (line 164-166 in the revised manuscript with track changes): “we performed GWAS for the phenotypic values of the eight plant architecture traits in each of the two environments (Y1, 2) and their BLUE values”.*

5. Line 168, “associated with the eight plant architecture traits” to “associated with at least one of the eight plant architecture traits”.

*Authors’ response 5: Thanks. We modified it accordingly (line 170 in the revised manuscript with track changes).*

6. I suggest placing the sentences “We took TEOSINTE BRANCHED1…, which is consistent with the previous finding.” in front of “Notably, the major loci for the length of main shoot …”.

*Authors’ response 6: Thanks. We modified it accordingly (line 180-195 in the revised manuscript with track changes).*

7. Please add more details to the Figure legends, especially for the Figure 4, so that readers can comprehend the findings simply from glancing at the Figures. “PL” to “Peduncle length (PL)” in line 436.

*Authors’ response 7: Thanks. We modified it accordingly (line 428-439 in the revised manuscript with track changes).*

8. Line 457, adjust the font size of “may”.

*Authors’ response 8: Thanks. We modified it accordingly.*

9. In the first two paragraphs of the “Manipulating main shoot length through peduncle” section, it is confused that 5,768 SNPs were identified as significantly associating with PL. Is it the truth? Or 5,768 SNPs were used to conduct the GWAS? If the original is right, why only four SNPs were studied furtherly? What about the remaining 5,764 SNPs?

*Authors’ response 9: Thanks. We provided the explanation accordingly.*

*The modifications in the manuscript (line 587-591 in the revised manuscript with track changes): “We identified one major locus related to the length of peduncle and main shoot. Within this major locus, four SNPs were significantly (−Log10[P-value]>5.0) associated with the traits and located in the gene TraesCS1A02G064800. Therefore, we identified TraesCS1A02G064800 as a candidate gene that controlled peduncle length. ” According to the suggestions, we moved this part to discussion.*

10. Line 564, the citation of “(Fig. 6b)” is not appropriate. Fig. 6b is just a LD block plot. I can’t get any gene information from it. And please label the SNPs so that the corresponding LD block can be recognized, such as connecting the target SNPs with the corresponding LD blocks using a line.

*Authors’ response 10: Thanks. We removed the citation of “(Fig. 6b)”.*

*The modifications in the manuscript (line 626-630 in the revised manuscript with track changes): “Local Manhattan plot (top) and LD heat map (bottom) surrounding the peak on chromosome 1A. Red color highlights the strong LD with the significant variants. Arrows indicate the four SNPs that were significantly (−Log10[P-value]>5.0) associated with the traits and located in the exonic region of this gene TraesCS1A02G064800. ”*

11. Figure 6f is not quoted in the manuscript. And the “Fig. 5g” in lines 591 and 593 incorrectly quoted.

*Authors’ response 11: Thanks. We quoted Fig. 6f and modified Fig. 5g accordingly (line 620 in the revised manuscript with track changes).*

12. In “Identification of putative selective sweeps” part, please clarify what software was used for the XP-CLR calculation and which population was used as the reference population.

*Authors’ response 12: Thanks. We clarified it accordingly.*

*The modifications in the manuscript (line 786-788 in the revised manuscript with track changes): “ The XP-CLR test [49] was used to detect selective sweeps to identify potential selective signals between Chinese cultivars (17 accessions) and landraces (42 accessions, reference population) in 306 word wheat accessions. ”*

13. Line 852, “…because the fd statistic value…” to “…because the negative fd statistic value…”

*Authors’ response 13: Thanks. We modified it accordingly (line 809 in the revised manuscript with track changes).*

14. Use italic format for the R package names.

*Authors’ response 14: Thanks. We modified it accordingly (line 699, 754, 758, 776, 782 in the revised manuscript with track changes).*

After going through the manuscript, remarks, and responses, I think the authors addressed the concerns from the reviewee #1 generally, except for the first question from reviewee #1:

1. "The multiple originations of wheat domestication have led to genetic and phenotypic variation in different regions worldwide. The 306 accessions were selected based on the genetic and phenotypic diversity across worldwide wheat accessions."

Performing population structure analyses, such as phylogenetic tree, or PCA, would be helpful to clarify the genetic diversity of the 306 accession rather than to prove the genetic and phenotypic variation by emphasizing the multiple originations and worldwide collection, which makes less sense for the question because even though they were from different geographic zones, they might underwent same selection bottleneck which led to the possible of convergent evolution.

*Authors’ response 1: Thanks for the kind suggestion. According to the suggestion, we performed principal component analysis (PCA) and phylogenetic tree (Additional file 2: Figure S1).*

*The modifications in the manuscript (line 115-126 in the revised manuscript with track changes): “To investigate the population differentiation of wheat accessions across the globe, we performed principal component analysis (PCA) and phylogenetic tree. The PCA result revealed that most varieties from Middle East, Europe and Asia could be distinguished by the first principal component (PC1), with an overall gradient from the Middle East to both Europe and Asia (Additional file 2: Figure S1a), which is consistent with the history of wheat dispersal [14, 15]. The neighbor-joining tree suggested that the 306 worldwide accessions could classify into some clades, which were associated with their geographical distribution (Additional file 2: Figure S1b). For instance, Asian accessions and some accessions from Middle East and Africa grouped into the same clade (Additional file 2: Figure S1b). Most European accessions clustered into one single clade (Additional file 2: Figure S1b).”*

*Figure S1. Phylogenetic relationships and population structures. a, Principal components analysis (PCA) plots of the first two components (PC1 and PC2) of 306 worldwide wheat accessions. The colors of dots indicate the population and location. b, Neighbor-joining (NJ) tree was built using a total of 40,710,923 SNPs. Branch colors reflect different populations. Accessions are arranged according to the populations and their geographic locations.*

2. "175 hard varieties and 96 soft varieties", which might be confusing for readers who are not studying wheats: hard and soft for what, seed?

*Authors’ response 2: Thanks for the kind suggestion. According to the suggestion, we clarified the hard and soft grain in Additional file 1: Table S1.*

*The clarification in Additional file 1: Table S1: “Hard and soft grains were used to distinguish the varieties of wheat by the hardness of their kernels. Grain hardness is one of the main quality traits of wheat and is associated with the properties of the Friabilin protein bound to the surface of the starch granules, which has a significant impact on wheat milling, baking, grading and final yield quality. (Reference: [1]Molecular genetics of puroindolines and related genes: regulation of expression, membrane binding properties and applications; [2] Molecular genetics of puroindolines and related genes: allelic diversity in wheat and other grasses.). Hard wheat is generally distinguished from soft wheat by the keratin content of the kernels. Hard wheat has a dense, translucent endosperm structure, also known as horniness or vitreousness, and when its horniness occupies more than 1/2 of its central cross-section, it is said to be a horny grain and is hard wheat. The endosperm of soft wheat is loose and gypsum-like, also known as powdery; in the case of wheat kernels, when less than 1/2 of the keratin is present, it is called powdery and soft wheat; when between 50% -70% of the keratin is present, it is called mixed. (Reference: [1]Molecular genetics of puroindolines and related genes: regulation of expression, membrane binding properties and applications; [2] Molecular genetics of puroindolines and related genes: allelic diversity in wheat and other grasses. [3]GB1351-99, Wheat National Standards[S]).*

3. " The 306 accessions included 96 winter varieties and 178 spring varieties, 175 hard varieties and 96 soft varieties, 179 landraces and 38 traditional cultivars". I don't think this statement is clear. It is easy to be misunderstood as 306=95+178+175+96+179+38. Winter/spring, soft/hard, and landrace/traditional cultivars are 3 inclusive categories. If the MS emphasized the domestication, it might be just using "The 306 accessions included 179 landraces and 38 traditional cultivars", the remaining information (hard/soft, winter/spring) can be referred to Table S1. Or any better expression patter.

*Authors’ response 3: Thanks for the kind suggestion. We clarified the statement accordingly.*

*The modifications in the manuscript (line 113-115 in the revised manuscript with track changes): “The 306 accessions included 179 landraces and 38 traditional cultivars (Additional file 1: Table S1). ”*

**Third round of review**

**Reviewer 2**

The manuscript (MS) has been improved a lot. But I still have two main severe concerns: 1. Regarding the track-back/provenance analysis of the Peduncle length, Length of the first, second, third and fourth internode, two pedigrees had been declared to use in the examination of the haplotype distribution in the wheat accessions at the beginning of this part. So, please provide the explanation if the claim is not arbitrary as a main part and finding of this MS: Can they represent all or dominant developments of Chinese wheat breeding? ---Actually, Hap^FIL-S^ was likely introduced from other founders which does not belong to the claimed two pedigrees as mentioned in the MS; 2. For the “Manipulating main shoot length through peduncle” part, I didn’t see any manipulation, and I don’t think the evidence were solid enough for the identification of the candidate gene *TraesCS1A02G064800*. Also, please use the near isogenic lines (NILs) of AA and GG to validate the phenotype to exclude the effect of the genetic background rather than RILs. If the author can’t provide the transgenic validation and NILs validation, it is better to move this part to discussion. Some detailed comments as follows:

1, In lines 42-43, “This enabled us to…” to “This makes it possible to…”.

2, In line 87, “has” to “have”.

3, In line 117, “phylogenetic tree” is not properly to some extent because it is just within wheat species even though I mentioned it in last comment. I would say “we performed population structure analyses”.

4, In line 126, add a blank between “).” and “The”.

5, In figure1C, the dendrogram in the Figure1c is unnecessary, or please explain it properly.

6, In lines 173-174, “…associated with the eight plant architecture traits” to “…associated with at least one of the eight plant architecture traits”

7, In line 227, add a blank in “that49%”

8, In lines 261-262, do these two pedigrees have specific name? I suggest: if have, please cite, otherwise, please name them in your MS.

9, In figure 3c, better to add the Y axis title.

10, In Figure 3e, the labels are not complete in x axis.

11, In line 285, “after 2020”? Is that right? Or “in 2020”?

12, In lines 291-292, “we used two important pedigrees in Chinese wheat breeding” to “we used two important pedigrees, xxx, and xxx, which are dominant in Chinese wheat breeding.” if the “dominant” is right.

13, In lines 483-484, “…this observed haplotype distribution explains …” to “…this observed haplotype distribution might explains …”. You can’t draw the conclusion with this kind of indirect evidence. I prefer to believe it is a discussion.

14, In lines 486-487, better to delete “four”. It is easy to confuse with “fourth”, which will be leading to a weird follow-up.

15, In line 545, “Four SNP”, please indicate them in Figures.

16, In line 563, “we introgressed the alleles to manipulate these two traits.” Where is the introgression manipulation?

**Authors Response**

**Point-by-point responses to the reviewers’ comments:**

Reviewer #2:

The manuscript (MS) has been improved a lot. But I still have two main severe concerns: 1. Regarding the track-back/provenance analysis of the Peduncle length, Length of the first, second, third and fourth internode, two pedigrees had been declared to use in the examination of the haplotype distribution in the wheat accessions at the beginning of this part. So, please provide the explanation if the claim is not arbitrary as a main part and finding of this MS: Can they represent all or dominant developments of Chinese wheat breeding? ---Actually, HapFIL-S was likely introduced from other founders which does not belong to the claimed two pedigrees as mentioned in the MS;

*Authors’ response 1: Thanks. According to the suggestion, we provided the explanation.*

*The modifications in the manuscript (line 290-296 in the revised manuscript with track changes): “To confirm the changes in frequency for the haplotypes at Y1-PL-1A-1, we used two important pedigrees (Aimengniu, Xiaoyan6) in Chinese wheat breeding. We examined the breeding histories of two important cultivars (Aimengniu, Xiaoyan6) that were initially released in the early 1980s. Aimengniu is a high yield variety, which is the founder genotype for more than 20 released Chinese cultivars, many of which are widely planted in China [9]. Xiaoyan6 was recognized through an award in 1985, and more than 40 Chinese cultivars have been derived from this founder genotype [9]. ”*

*The modifications in the manuscript (line 326-329 in the revised manuscript with track changes): “This finding was consistent with reduced plant height in wheat breeding. However, we cannot exclude the possibility that HapFIL-S was likely introduced from other founders which does not belong to these two pedigrees. ”*

2. For the “Manipulating main shoot length through peduncle” part, I didn’t see any manipulation, and I don’t think the evidence were solid enough for the identification of the candidate gene TraesCS1A02G064800. Also, please use the near isogenic lines (NILs) of AA and GG to validate the phenotype to exclude the effect of the genetic background rather than RILs. If the author can’t provide the transgenic validation and NILs validation, it is better to move this part to discussion.

*Authors’ response 2: Thanks. According to the suggestion, we moved this part to discussion. The modifications in the manuscript (line 587-642 in the revised manuscript with track changes).*

Some detailed comments as follows:

1, In lines 42-43, “This enabled us to…” to “This makes it possible to…”.

*Authors’ response 3: Thanks. We modified it accordingly (line 42-43 in the revised manuscript with track changes).*

2, In line 87, “has” to “have”.

*Authors’ response 4: Thanks. We modified it accordingly (line 87 in the revised manuscript with track changes).*

3, In line 117, “phylogenetic tree” is not properly to some extent because it is just within wheat species even though I mentioned it in last comment. I would say “we performed population structure analyses”.

*Authors’ response 5: Thanks. We modified it accordingly (line 113-114 in the revised manuscript with track changes).*

4, In line 126, add a blank between “).” and “The”.

*Authors’ response 6: Thanks. We modified it accordingly (line 122 in the revised manuscript with track changes).*

5, In figure1C, the dendrogram in the Figure1c is unnecessary, or please explain it properly.

*Authors’ response 7: Thanks. We removed the dendrogram in the Fig. 1c according to the suggestion.*

6, In lines 173-174, “…associated with the eight plant architecture traits” to “…associated with at least one of the eight plant architecture traits”

*Authors’ response 8: Thanks. We modified it accordingly (line 170 in the revised manuscript with track changes).*

7, In line 227, add a blank in “that49%”

*Authors’ response 9: Thanks. We modified it accordingly (line 225 in the revised manuscript with track changes).*

8, In lines 261-262, do these two pedigrees have specific name? I suggest: if have, please cite, otherwise, please name them in your MS.

*Authors’ response 10: Thanks. We named them according to the suggestion.*

*The modifications in the manuscript (line 290-291 in the revised manuscript with track changes): “ To confirm the changes in frequency for the haplotypes at Y1-PL-1A-1, we used two important pedigrees (Aimengniu, Xiaoyan6) in Chinese wheat breeding. ”*

9, In figure 3c, better to add the Y axis title.

*Authors’ response 11: We added the Y axis title according to the suggestion.*

10, In Figure 3e, the labels are not complete in x axis.

*Authors’ response 12: We modified the labels in x axis in Fig. 3e.*

11, In line 285, “after 2020”? Is that right? Or “in 2020”?

*Authors’ response 13: Thanks. We modified it accordingly.*

*The modifications in the manuscript (line 283-284 in the revised manuscript with track changes): “ whose relative frequency increased from 41% before 1960 to 75% in the time window of 2001-2020.”*

12, In lines 291-292, “we used two important pedigrees in Chinese wheat breeding” to “we used two important pedigrees, xxx, and xxx, which are dominant in Chinese wheat breeding.” if the “dominant” is right.

*Authors’ response 14: Thanks. We modified it accordingly.*

*The modifications in the manuscript (line 290-296 in the revised manuscript with track changes): “To confirm the changes in frequency for the haplotypes at Y1-PL-1A-1, we used two important pedigrees (Aimengniu, Xiaoyan6) in Chinese wheat breeding. We examined the breeding histories of two important cultivars (Aimengniu, Xiaoyan6) that were initially released in the early 1980s. Aimengniu is a high yield variety, which is the founder genotype for more than 20 released Chinese cultivars, many of which are widely planted in China [9]. Xiaoyan6 was recognized through an award in 1985, and more than 40 Chinese cultivars have been derived from this founder genotype [9]. ”*

13, In lines 483-484, “…this observed haplotype distribution explains …” to “…this observed haplotype distribution might explains …”. You can’t draw the conclusion with this kind of indirect evidence. I prefer to believe it is a discussion.

*Authors’ response 15: We modified it accordingly (line 493 in the revised manuscript with track changes).*

14, In lines 486-487, better to delete “four”. It is easy to confuse with “fourth”, which will be leading to a weird follow-up.

*Authors’ response 16: We deleted “four” accordingly (line 497 in the revised manuscript with track changes).*

15, In line 545, “Four SNP”, please indicate them in Figures.

*Authors’ response 17: We indicated the four SNPs in Fig. 6b.*

*The modifications in the manuscript (line 628-630 in the revised manuscript with track changes): “Arrows indicate the four SNPs that were significantly (−Log10[P-value]>5.0) associated with the traits and located in the gene TraesCS1A02G064800. ”*

16, In line 563, “we introgressed the alleles to manipulate these two traits.” Where is the introgression manipulation?

*Authors’ response 18: Thanks. We modified it accordingly.*

*The modifications in the manuscript (line 601-603 in the revised manuscript with track changes): “we assessed the phenotypic differences of these two traits between the alleles in a genetic population. ”*

**Fourth round of review**

**Reviewer 2**

I think the manuscript is good to accept after making a little improvement of the Fig6b: 1, the dots are blurry which makes the colors of different dots hard to distinguish；2，the arrows are not easy to tell neither---try to select slim arrows and different color with apparent contrast; 3, link the four important signals/dots with their corresponding LD block directly, which makes them easy to recognize, rather than indicating their LD from the position, which is hard to locate them.
